# Functional variation in phyllogen, a phyllody‐inducing phytoplasma effector family, attributable to a single amino acid polymorphism

**DOI:** 10.1111/mpp.12981

**Published:** 2020-08-19

**Authors:** Nozomu Iwabuchi, Yugo Kitazawa, Kensaku Maejima, Hiroaki Koinuma, Akio Miyazaki, Ouki Matsumoto, Takumi Suzuki, Takamichi Nijo, Kenro Oshima, Shigetou Namba, Yasuyuki Yamaji

**Affiliations:** ^1^ Department of Agricultural and Environmental Biology Graduate School of Agricultural and Life Sciences The University of Tokyo Tokyo Japan; ^2^ Faculty of Bioscience Hosei University Tokyo Japan

**Keywords:** horizontal gene transfer, MADS domain transcription factors, natural variation, phyllody, phyllogen, phytoplasma, α‐helix

## Abstract

Flower malformation represented by phyllody is a common symptom of phytoplasma infection induced by a novel family of phytoplasma effectors called phyllogens. Despite the accumulation of functional and structural phyllogen information, the molecular mechanisms of phyllody have not yet been integrated with their evolutionary aspects due to the limited data on their homologs across diverse phytoplasma lineages. Here, we developed a novel universal PCR‐based approach to identify 25 phytoplasma phyllogens related to nine “*Candidatus* Phytoplasma” species, including four species whose phyllogens have not yet been identified. Phylogenetic analyses showed that the phyllogen family consists of four groups (phyl‐A, ‐B, ‐C, and ‐D) and that the evolutionary relationships of phyllogens were significantly distinct from those of phytoplasmas, suggesting that phyllogens were transferred horizontally among phytoplasma strains and species. Although phyllogens belonging to the phyl‐A, ‐C, and ‐D groups induced phyllody, the phyl‐B group lacked the ability to induce phyllody. Comparative functional analyses of phyllogens revealed that a single amino acid polymorphism in phyl‐B group phyllogens prevented interactions between phyllogens and A‐ and E‐class MADS domain transcription factors (MTFs), resulting in the inability to degrade several MTFs and induce phyllody. Our finding of natural variation in the function of phytoplasma effectors provides new insights into molecular mechanisms underlying the aetiology of phytoplasma diseases.

## Graphical abstract

Comparison of phyllogen, a phyllody‐inducing effector family, revealed its molecular evolution and functional variation attributed to a single amino acid polymorphism governing the phyllody symptoms of phytoplasma infection.

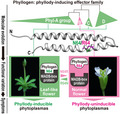

## 1 INTRODUCTION

Phytoplasmas (“*Candidatus Phytoplasma*” spp.) are obligate intracellular plant pathogens in the class Mollicutes. They are transmitted by insect vectors such as leafhoppers, planthoppers, and psyllids (Namba, 2019), and can infect more than 1,000 plant species worldwide (Marcone, 2014). Phytoplasma‐infected plants exhibit a wide range of unique symptoms, including flower malformation, yellowing, dwarfing, witches’ broom, purple tops, and phloem necrosis (Namba, 2019). Although the molecular mechanisms behind these symptoms are not fully understood, several secreted proteins of phytoplasmas, called effectors, can induce these symptoms (Hoshi *et al*., 2009; Sugio *et al*., 2011; Maejima *et al*., 2014; Minato *et al*., 2014).

Flower malformation, such as phyllody (floral organs turning into leaf‐like structures), virescence (green colouration of floral organs), and a loss of floral meristem determinacy (the production of stem‐like pistils), is a common symptom of phytoplasma infection (Chaturvedi *et al*., 2010; Musetti and Pagliari, 2019). Recently, it was reported that a novel gene family of phytoplasma effectors, designated phyllody‐inducing genes or the phyllogen family, induces flower malformation in several eudicots (MacLean *et al*., 2011; Maejima *et al*., 2014; Yang *et al*., 2015; Kitazawa *et al*., 2017). Phyllogens target the products of floral homeotic genes that constitute the floral quartet model (MacLean *et al*., 2014; Maejima *et al*., 2014), which in turn encode MADS domain transcription factors (MTFs) that are divided into four classes functionally: A, B, C, and E (Smaczniak *et al*., 2012). Phyllogens recognize A‐ and E‐class MTFs of angiosperms and degrade them in a proteasome‐dependent manner (MacLean *et al*., 2014; Maejima *et al*., 2014, 2015; Kitazawa *et al*., 2017). Additionally, SAP54, a phyllogen of “*Ca*. P. asteris” AY‐WB strain, interacts with two isoforms of the radiation sensitive 23 (RAD23) family, RAD23C/D (MacLean *et al*., 2014). RAD23C/D are substrate shuttle factors that can transfer ubiquitinated proteins to the 26S proteasome (Farmer *et al*., 2010) and are essential for phyllogen‐mediated phyllody induction (MacLean *et al*., 2014). Recent crystal structure analyses have revealed that the K domain of MTFs, a phyllogen‐binding region (MacLean *et al*., 2014), has two α‐helices with conserved hydrophobic residues that are important for the tetramerization of MTFs (Puranik *et al*., 2014). Two phyllogens (PHYL1_OY_ and PHYL1_PnWB_, phyllogens of “*Ca*. P. asteris” OY and “*Ca*. P. aurantifolia” PnWB strains, respectively) have similar structures based on two α‐helices that are important for phyllody‐inducing‐activity (Iwabuchi *et al*., 2019; Liao *et al*., 2019). Thus, the function and structure of a couple of phyllogens have been reported; however, the molecular mechanisms of phyllody have not yet been integrated with evolutionary aspects due to the limited data on their homologs across diverse phytoplasma lineages.

The accumulation of phytoplasma genome information has enabled the identification of phyllogens from seven “*Ca*. Phytoplasma” species (Chung *et al*., 2013; Maejima *et al*., 2014; Mitrović *et al*., 2014; Wang *et al*., 2018a; Fernández *et al*., 2019), while phyllogens have not yet been found in several phytoplasmas that induce phyllody symptoms in their host plants, such as “*Ca*. P. japonicum” (Arashida *et al*., 2008b). Phyllogen genes have been identified using two approaches: PCR using specific primers targeting up‐ and downstream regions of phyllogens and whole‐ or draft‐genome sequencing. The former approach is based on the fact that several phyllogens have been found around the clusters of repeated gene sequences, namely, potential mobile units (PMUs) (Jomantiene *et al*., 2007; Arashida *et al*., 2008a; Sugio and Hogenhout, 2012). Phyllogens sharing highly conserved sequences with known phyllogen genes, such as *PHYL1_OY_*, were identified in 17 strains related to four different “*Ca*. Phytoplasma” species using a PCR‐based method (Maejima *et al*., 2014; Fernández *et al*., 2019). An alternative approach, whole‐ or draft‐genome sequencing, could identify phyllogens unable to be detected with PCR‐based methods. The phyllogen gene *PHYL1_PnWB_*, identified using this approach, was not located near PMUs (Chung *et al*., 2013). Nevertheless, no phyllogens have been found in draft‐genome sequences of “*Ca*. P. phoenicium”, “*Ca*. P. pruni”, or “*Ca*. P. oryzae” (Lee *et al*., 2015; Quaglino *et al*., 2015; Fischer *et al*., 2016), possibly due to the difficulty in determining the complete genome sequences of phytoplasmas. Therefore, more universal and efficient means to identify phyllogen genes from a variety of phytoplasmas are required.

Here, we report a novel approach to identifying diverse phyllogens by focusing on two amino acid regions conserved in the gene family. Phylogenetic analyses based on determined phyllogen gene sequences strongly suggest that phyllogen genes are horizontally transferred among phytoplasmas. By comparing variation in natural function among members of the phyllogen family, we found that a group of phyllogens lacks the ability to induce phyllody and identified a polymorphic residue that is essential for phyllody‐inducing activity as well as the degradation of A‐ and E‐class MTFs.

## 2 RESULTS

### 2.1 Identification of phyllogens with universal PCR and genome walking

Multiple sequence alignment of three phyllogen proteins (PHYL1_OY_, PHYL1_PnWB_, and SAP54) showed the presence of two conserved amino acid regions within the secreted part (Figure S2a). We designed a degenerate primer pair (PHYL‐F/R) on these conserved regions that also matched all other known phyllogens (Figure S3). PCR using the primer pair resulted in the amplification of DNA fragments ranging from 177 to 210 bp from the genomic DNA of 25 phytoplasma strains (Figure S2c; indicated as “this study” in Table S1). These strains were related to nine species, including four species (“*Ca*. P. fragariae”, “*Ca*. P. fraxini”, “*Ca*. P. japonicum”, and “*Ca*. P. oryzae”) whose phyllogen genes have not been reported (Table S1). Each amplicon shared >70% nucleotide sequence identity with at least one of the known phyllogen genes, which suggests that each of the amplified DNA fragments is part of a phyllogen (Table S3). We performed genome walking for nine strains related to seven species (“*Ca*. P. asteris” HP and PvWB, “*Ca*. P. aurantifolia” FBP and WBDL, “*Ca*. P. fragariae” SY, “*Ca*. P. fraxini” ASHy2, “*Ca*. P. japonicum” JHP, “*Ca*. P. oryzae” RYD, and “*Ca*. P. ziziphi” JWB strains) to determine the up‐ and downstream sequences of each PCR amplicon (Figure S2b). The 202–1,358 bp of upstream sequences and 464–1,508 bp of downstream sequences obtained were assembled with the corresponding sequences between primers PHYL‐F/R. The validity of each assembled sequence was examined with PCR amplification using a primer pair designed on the up‐ and downstream sequences (Figure S2b), which resulted in the amplification of a DNA fragment of appropriate size (Figure S2d). In each assembled sequence, a putative ribosome‐binding site (5′‐AAGGAG‐3′; Berg and Seemüller, 1999) and a start and in‐frame stop codon were found at almost the same positions as the known phyllogens. Thus, we identified nine new full‐length phyllogen genes. Several phyllogens, such as the one from the ASHy2 strain (PHYL1_ASHy2_), lost their α‐helix structure (indicated by the symbol ^Ψ^ in Table S1 and Figure S3) because of an additional premature stop codon.

**FIGURE 1:**
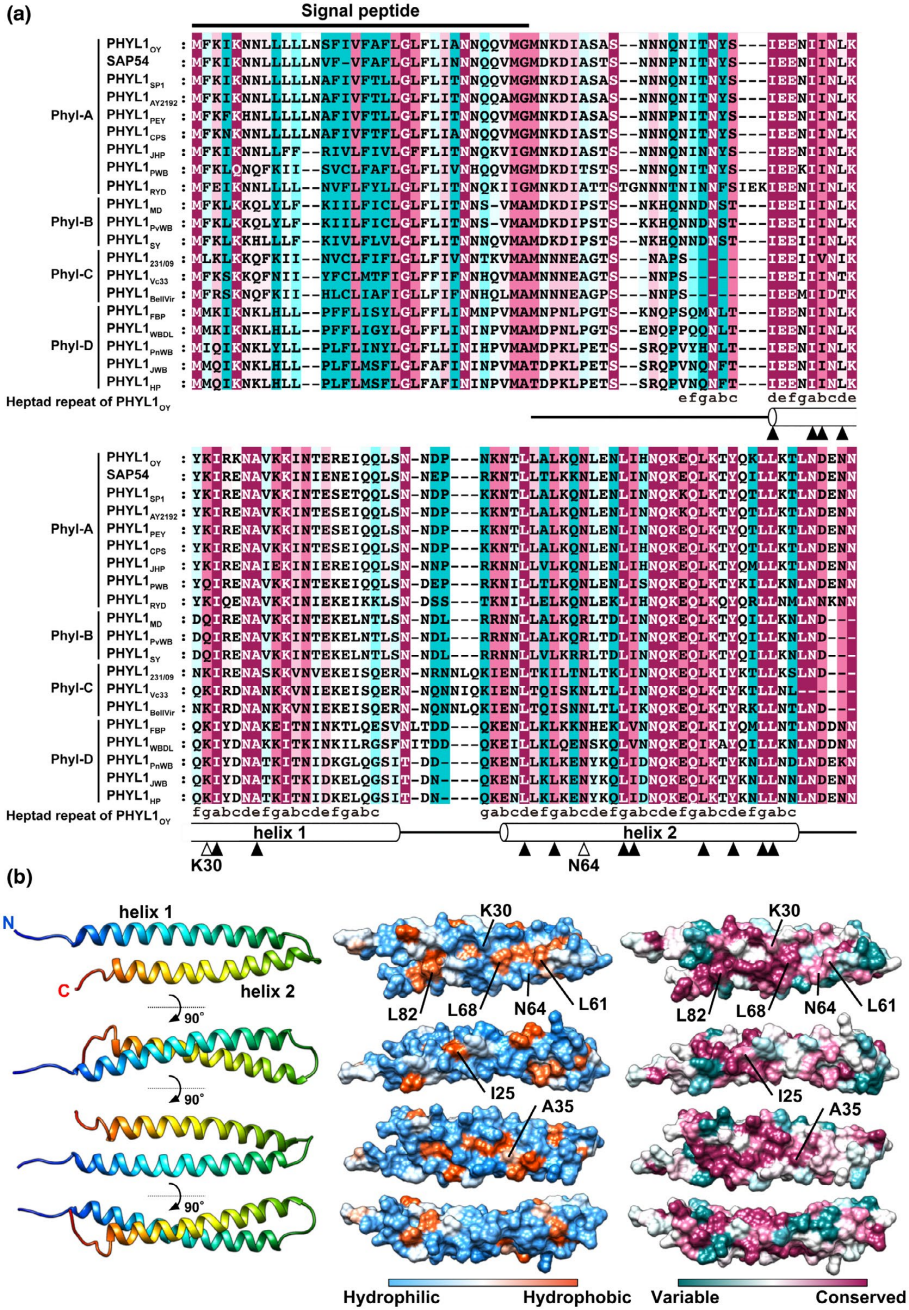
Amino acid conservation and structural properties of phyllogens. (a) Alignment of the full‐length protein sequences of the phyllogen family. Phyllogen protein sequences were aligned using the MUSCLE algorithm. Consensus secondary structure elements of phyllogens predicted by PROMALS3D are depicted below. Open boxes represent α‐helices. Filled and open arrowheads indicate conserved hydrophobic residues and polymorphic residues between phyl‐B and the other groups (K30 and N64, numbering based on PHYL1_OY_ excluding signal peptides), respectively. Sequence conservation calculations were performed with ConSurf (Ashkenazy *et al*., 2016). Conservation scores range from cyan (not conserved) to white (average) and to magenta (highly conserved). (b) Surface structure properties of PHYL1_OY_. Overall view of the ribbon diagram (left), hydrophobicity surface (middle), and conserved structure surface (right) of PHYL1_OY_ (PDB ID: 6JQA, residues 7–91 of subunit A without iodine atoms). Hydrophobicity scores range from blue (mostly hydrophilic) to white (average) and to orange red (mostly hydrophobic). Conservation scores range as (a)

A putative signal peptide cleavage site and two consensus α‐helices were predicted at the same location in each phyllogen (Figure 1a). Hydrophobic residues in the two α‐helical regions, which are important for phyllody‐inducing activity (Iwabuchi *et al*., 2019; Liao *et al*., 2019), were highly conserved among diverse phyllogens identified in this and previous studies (Figures 1a and S4; positions a, b, d, and e on helix 1 and positions a, d, and g on helix 2). The conserved hydrophobic residues at positions a and d on each helix (I20, I24, L27, I31, L58, I69, L76, and L83; numbering based on PHYL1_OY_ excluding signal peptides) were oriented toward the interiors of both α‐helices (Figure S4). In contrast, the conserved hydrophobic residues at positions b and e on helix 1 (I25 and A35) and position g on helix 2 (L61, L68, and L82), which are suggested to be available to interact with host factors (Liao *et al*., 2019), are exposed on the protein surface (Figures 1b and S4). These results suggest that the conserved hydrophobic residues support protein structural integrity and the ability to interact with host factors of phyllogen.

### 2.2 Evolutionary and structural relationships in the phyllogen family

**FIGURE 2:**
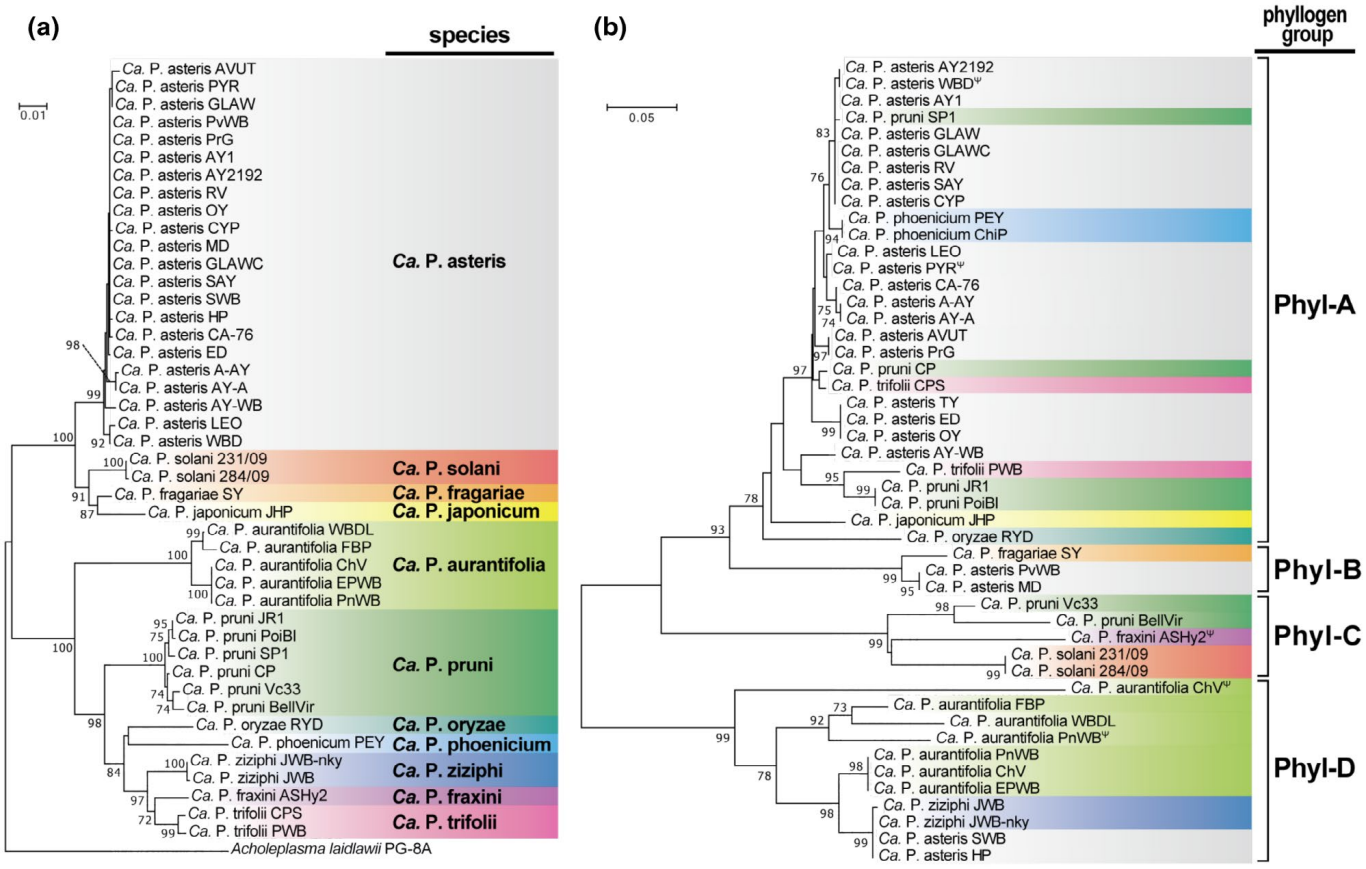
Phylogenetic comparison of the phyllogen family. (a) and (b) Neighbour‐joining phylogenetic tree based on 16S rRNA between primers SN910601/SN910502 (a) and full‐length phyllogen (b) gene nucleotide sequences. Sequences were aligned with the MUSCLE multiple alignment algorithm and analysed with a complete‐deletion option. *Acholeplasma laidlawii* strain PG‐8A was used to root the tree of 16S rRNA. Numbers at the nodes represent the percentage of bootstrap values obtained for 1,000 replicates (only values >70% are shown). Bars indicate the number of nucleotide substitutions per site. For the C‐terminus truncated phyllogens due to the premature stop codon (indicated by ^Ψ^), the nucleotide regions after the premature stop codons were also included in the MUSCLE alignment. Full strain names and GenBank accession numbers are listed in Table S1. Background colours define related “*Candidatus* Phytoplasma” species

Pairwise sequence comparisons showed considerable sequence variation among the phyllogen family, with >66.4% and >40.9% sequence identity at the nucleotide and amino acid levels, respectively (Table S4). To investigate the evolutionary relationship within the gene family, we performed a phylogenetic comparison on phyllogen and the 16S rRNA gene representing the phylogeny of phytoplasmas. The neighbour‐joining tree using 43 full‐length phyllogen nucleotide sequences indicated that the phyllogen family can be divided into four distinct groups supported with high bootstrap values (78%, 99%, 99%, and 99% for phyl‐A, ‐B, ‐C, and ‐D, respectively; Figure 2b). Interestingly, the tree topology of the phyllogen gene was clearly different from that of the 16S rRNA gene; each phyllogen group consisted of diverse “*Ca*. Phytoplasma” species (Figure 2a). Phyllogens of “*Ca*. P. asteris” were included in three different groups—phyl‐B (MD and PvWB strains), phyl‐D (HP and SWB strains), and phyl‐A (the other strains)—and phyllogens of “*Ca*. P. pruni” were included in both phyl‐C (BellVir and Vc33 strains) and phyl‐A (the other strains; Figure 2b). Similar results were obtained from the tree topology of the phyllogen gene based on partial nucleotide sequences between the PHYL‐F/R regions of all strains (Figure S5). These results indicate that the phyllogen family has a different evolutionary history from the 16S rRNA gene.

Structural modelling of PHYL1_SY_ and PHYL1_231/09_ belonging to the phyl‐B and ‐C groups, respectively, was performed based on the PHYL1_OY_ structure (PDB ID: 6JQA). The resulting model (C‐scores of 0.21 and −0.08 for PHYL1_SY_ and PHYL1_231/09_, respectively) also contained a coiled‐coil structure with a hydrophobic surface similar to that of phyl‐A or ‐D groups (Figure S6), which also supports the functional importance of the conserved hydrophobic residues.

### 2.3 The phyl‐B group does not induce phyllody

**FIGURE 3:**
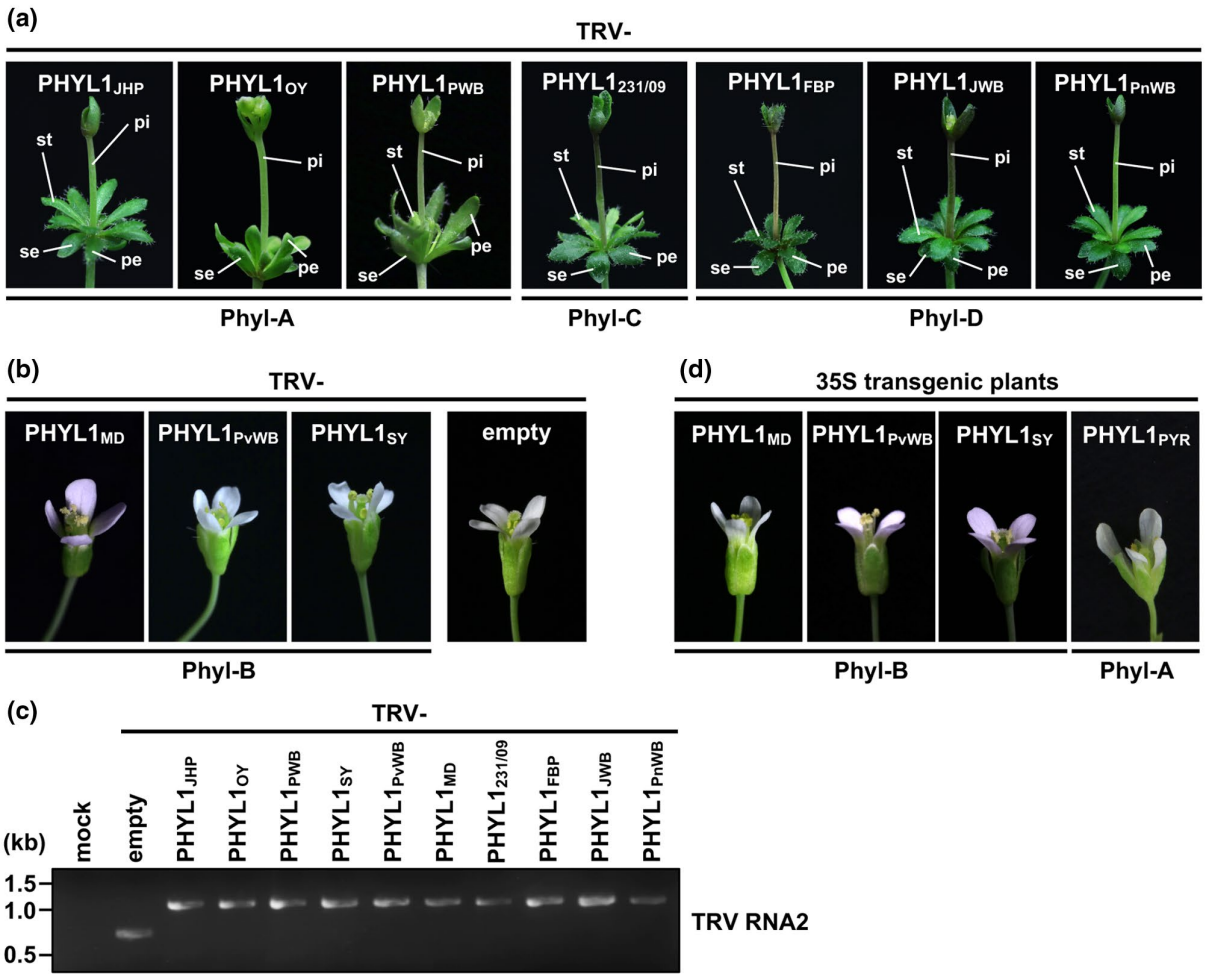
Phyllody‐inducing activity differs among phyllogen groups. (a) and (b) Floral phenotypes of *Arabidopsis thaliana* plants infected with the tobacco rattle virus (TRV) vector carrying phyllogens belonging to either the phyl‐A, ‐C, or ‐D group (a) or the phyl‐B group (b). Phyllody‐like phenotypes consisted of leaf‐like sepals (se), leaf‐like petals (pe), leaf‐like stamens (st), and a stem‐like pistil (pi). (c) Confirmation of the infection and insertion stability of the TRV vector by reverse transcription (RT)‐PCR. RT‐PCR was performed with primers flanking the site of insertion in RNA2 of the virus about 30 days after inoculation in *A*. *thaliana* plants. (d) Floral phenotypes of the phyl‐B group‐ and α‐helix truncated phyllogen (phyl‐A: PHYL1_PYR_)‐overexpressing transgenic *A*. *thaliana* plants

**FIGURE 4:**
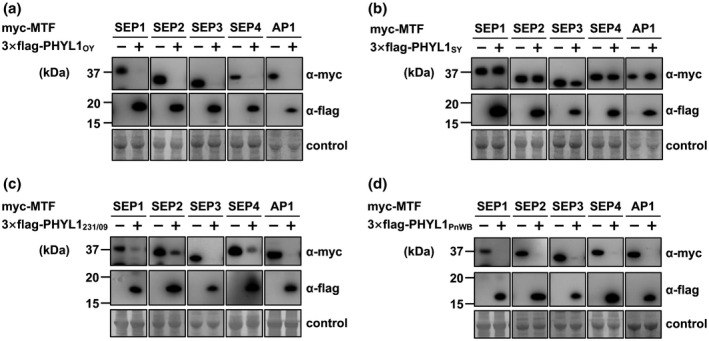
PHYL1_SY_ has little activity in the degradation of MADS domain transcription factors (MTFs). (a)–(d) Accumulation of transiently expressed *Arabidopsis* A‐ and E‐class MTFs upon co‐expression of phyllogens. *Agrobacterium* cultures (OD_600_ = 1.0) expressing P19, Myc‐fused MTFs (SEP1–4 and AP1), and either 3 × FLAG‐fused PHYL1s (phyl‐A: PHYL1_OY_ [a], phyl‐B: PHYL1_SY_ [b], phyl‐C: PHYL1_231/09_ [c], or phyl‐D: PHYL1_PnWB_ [d]) were mixed at a ratio of 1:10:1 and infiltrated into *Nicotiana benthamiana* leaves. Accumulation of Myc‐ or FLAG‐fused proteins was evaluated 36 hr after infiltration by immunoblotting using an anti‐Myc (α‐myc) or anti‐FLAG (α‐flag) antibody. Coomassie brilliant blue‐stained membranes are shown as a loading control

Three phyllogens belonging to either phyl‐A (PHYL1_OY_ and SAP54) or ‐D (PHYL1_PnWB_) induce phyllody in *Arabidopsis thaliana* (MacLean *et al*., 2011; Maejima *et al*., 2014; Yang *et al*., 2015). To test whether phyllody‐inducing activity is conserved across the gene family, we selected for further study a variety of phyllogens of each group (phyl‐A: *PHYL1_JHP_* and *PHYL1_PWB_*; phyl‐B: *PHYL1_MD_*, *PHYL1_PvWB_*, and *PHYL1_SY_*; phyl‐C: *PHYL1_231/09_*; phyl‐D: *PHYL1_FBP_* and *PHYL1_JWB_*) in addition to *PHYL1_OY_* and *PHYL1_PnWB_*. Each phyllogen was expressed in *A*. *thaliana* using the tobacco rattle virus (TRV) vector as described by Iwabuchi *et al*. (2019). Then, 20–30 days after virus inoculation, *A*. *thaliana* plants infected with TRV‐PHYL1_JHP_, ‐PHYL1_PWB_, ‐PHYL1_231/09_, ‐PHYL1_JWB_, ‐PHYL1_FBP_, or ‐PHYL1_PnWB_ showed almost the same phyllody phenotype as described previously (Figure 3a; Iwabuchi *et al*., 2019). Sepals, petals, and stamens were converted into enlarged leaf‐like structures covered with stellate trichomes, and the pistil reverted to a stem‐like structure with secondary flowers at the top as well as a loss of floral meristem determinacy (Figure 3a). Furthermore, the transient co‐expression assay in *Nicotiana benthamiana* showed that PHYL1_231/09_ and PHYL1_PnWB_ significantly decreased the amount of SEP1–4 or AP1 as in the case of PHYL1_OY_ (Figure 4a,c,d; Maejima *et al*., 2014, 2015).

In contrast, phyllogens belonging to phyl‐B (PHYL1_MD_, PHYL1_PvWB_, and PHYL1_SY_) induced no flower malformation in *A*. *thaliana* when expressed from the TRV vector (Figure 3b). Successful infection of the TRV vector and inserted sequence stability of each phyllogen gene therein were confirmed by reverse transcription (RT)‐PCR using pTRV2‐specific primers (Figure 3c). Floral organs in the 35S::*PHYL1_MD_*, 35S::*PHYL1_PvWB_*, and 35S::*PHYL1_SY_* transgenic *A*. *thaliana* also showed no malformation (Figure 3d). Similar results were obtained in flowers of *N*. *benthamiana* infected with TRV vector expressing PHYL1_MD_, PHYL1_PvWB_, or PHYL1_SY_ (Figure S7). Because the non‐phyllody‐inducing phyllogens belonging to the phyl‐B group were almost identical (>92.4% at amino acid level), PHYL1_SY_ was used to evaluate floral MTF‐degradation activity in planta. Compared to PHYL1_OY_, PHYL1_SY_ did not significantly decrease the amount of SEP1–4 or AP1 (Figure 4b).

In addition, we tested the phyllody‐inducing activity of natural α‐helix‐truncated phyllogens due to early stop codons (Figure S3) using PHYL1_PYR_, which belongs to the phyl‐A group and does not induce degradation of SEP3 (Maejima *et al*., 2014). Floral organs in the 35S::*PHYL1_PYR_* transgenic *A*. *thaliana* showed no malformation (Figure 3d), in accordance with previous reports indicating the functional importance of the α‐helix (Iwabuchi *et al*., 2019; Liao *et al*., 2019).

### 2.4 Interaction specificity of the phyllogen family with A‐ and E‐class MTFs and RAD23

To determine the host factors involved in the loss of phyllody‐inducing activity in the phyl‐B group, we compared the interaction of phyllogens with *Arabidopsis* A‐ and E‐class MTFs and RAD23C/D using yeast two‐hybrid (Y2H) analyses (Table 1 and Figure S10). Phyllody‐inducing phyllogens (phyl‐A: PHYL1_PWB_, phyl‐C: PHYL1_231/09_, phyl‐D: PHYL1_FBP_, and PHYL1_JWB_) interacted with AP1 (A‐class), SEP1–4 (E‐class), and RAD23C/D to the same extent as PHYL1_OY_ and PHYL1_PnWB_. Among non‐phyllody‐inducing phyllogens, PHYL1_PYR_ failed to interact with AP1, SEP1, SEP2, SEP4, and RAD23C/D as well as SEP3 (Maejima *et al*., 2014). These results indicate that PHYL1_PYR_ lost phyllody‐inducing activity because of the loss of interaction with these host factors. However, the phyl‐B group (PHYL1_MD_, PHYL1_PvWB_, and PHYL1_SY_) interacted with SEP1 and RAD23C/D to the same extent as the other phyllogens, although no interactions were observed with SEP4, and weak interactions were observed with AP1, SEP2, and SEP3. These results suggest that the phyl‐B group has a novel type of natural loss of phyllody‐inducing mutants due to changes in interaction specificity with MTFs.

**TABLE 1:** Interaction specificity with floral MADS domain transcription factors and RAD23 protein in yeast cells

Phyllogen group	DNA‐binding domain (bait)	Activation domain (prey)							
		Empty	SEP1	SEP2	SEP3	SEP4	AP1	RAD23C	RAD23D
	Empty	−	−^a^	−^a^	−^b^	−^a^	−^b^	−^a^	−
Phyl‐A	PHYL1_OY_	−^a^	+++^a^	+++^a^	+++^a^	+++^a^	++	++^a^	++
	PHYL1_PWB_	−	++	++	++	++	++	++	++
	PHYL1_PYR_ ^Ψ^	−^b^	−	−	−^b^	−	−	−	−
Phyl‐B	PHYL1_MD_	−	++	+	+	−	+	++	++
	PHYL1_PvWB_	−	++	+	+	−	+	++	++
	PHYL1_SY_	−	++	+	+	−	+	++	++
	PHYL1_SY_^R64N^	−	++	++	++	+	++	++	++
	PHYL1_SY_^Q30K/R64N^	−	++	++	++	++	++	++	++
Phyl‐C	PHYL1_231/09_	−	++	++	++	++	++	++	++
Phyl‐D	PHYL1_FBP_	−	++	++	++	++	++	++	++
	PHYL1_JWB_	−	++	++	++	++	++	++	++
	PHYL1_PnWB_	−	++	++	++^c^	++	++	++	++

Note

+++ The yeast grew on all media; ++ the yeast grew on − LWH+3AT, −LWH, and − LW; + the yeast grew on − LWH and − LW; − the yeast grew only on − LW.

^Ψ^ a C‐terminus truncated mutant due to a premature stop codon. Several results were previously reported in ^a^ Iwabuchi *et al. *(2019), ^b^ Maejima *et al. *(2014), or ^c^ Kitazawa *et al. *(2017).

### 2.5 Polymorphic residues at position 64 in phyllogens responsible for phyllody induction

**FIGURE 5:**
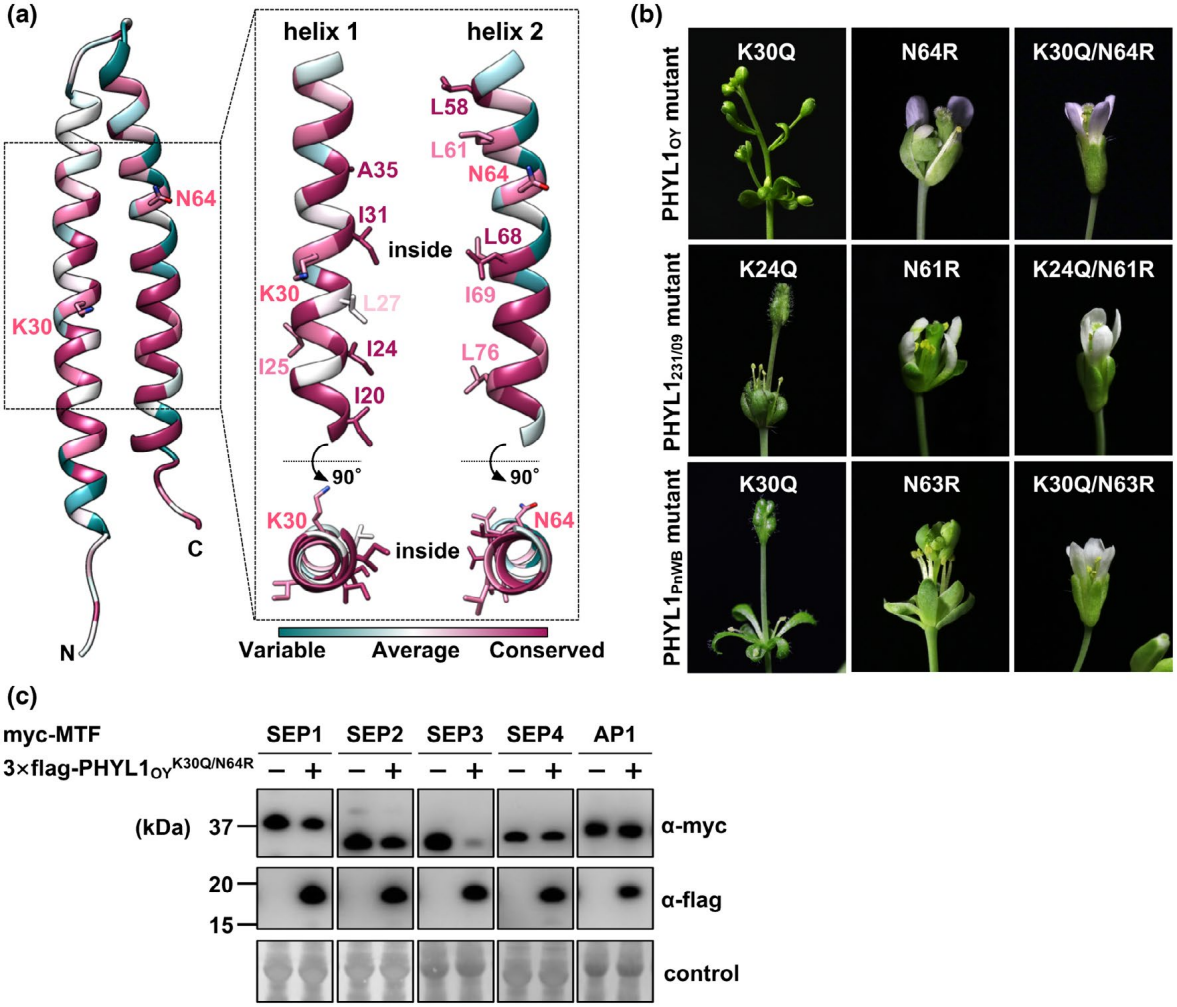
Substitution of conserved N64 residue abolishes the phyllody‐inducing activity of phyllogens. (a) Positions of polymorphic residues on ribbon representation of the crystal structure of PHYL1_OY_. Two hydrophilic residues (K30 and N64) that mutated to those of the phyl‐B group (Q30 and R64) and conserved hydrophobic residues such as leucine (L) and isoleucine (I) indicated in Figure 1a are shown as sticks. Conservation scores are mapped as in Figure 1b. (b) Floral phenotypes of *Arabidopsis thaliana* plants infected with tobacco rattle virus (TRV) vector carrying PHYL1_OY_ K30Q and/or N64R mutants and PHYL1_231/09_ and PHYL1_PnWB_ mutants of corresponding K and N residues (K24 and N61 for PHYL1_231/09_ and K30 and N63 for PHYL1_PnWB_, respectively). (c) Accumulation of transiently expressed Myc‐fused *Arabidopsis* floral MADS domain transcription factors (MTFs) upon co‐expression with 3 × FLAG‐fused PHYL1_OY_^K30Q/N64R^. In planta protein expression and detection were performed as described in Figure 4

To elucidate the amino acid residue(s) involved in the functional variation among phyllogen groups, we compared their sequences. Two hydrophilic residues, lysine at position 30 (K30) and asparagine at position 64 (N64), were conserved among the phyl‐A, ‐C, and ‐D groups with one exception: PHYL1_PWB_, belonging to phyl‐A with glutamine at position 30 (Q30), but not in all members of phyl‐B (Q30 and arginine at position 64 [R64]; Figures 1a and S8; open arrowhead symbol). Mapping of both residues onto the PHYL1_OY_ structure revealed that they were exposed on the same side of the PHYL1_OY_ protein surface, unlike conserved hydrophobic residues at positions a and d, which are oriented toward the interiors of both α‐helices (Figures 1b, 5a and S4). To test whether K30 and N64 are important for phyllody‐inducing activity, we introduced reciprocal substitutions at positions 30 and 64 to those of PHYL1_SY_ into PHYL1_OY_. PHYL1_OY_^K30Q^ and PHYL1_OY_ both induced severe flower malformation (Figures 3a and 5b). In contrast, PHYL1_OY_^N64R^ induced moderate flower malformation, such as asymmetry of petals, but not phyllody. Moreover, PHYL1_OY_^K30Q/N64R^ did not induce any flower malformation (Figure 5b), as in the case of the phyl‐B group. Similar results were obtained when mutations of corresponding K and N residues were introduced into PHYL1_231/09_ and PHYL1_PnWB_ belonging to phyl‐C and phyl‐D, respectively (Figure 5b). Western blotting analyses showed that PHYL1_OY_^K30Q/N64R^ did not decrease the amount of each *Arabidopsis* MTF compared to PHYL1_OY_ (Figure 5c). These results suggest that N64 and K30 play critical and marginal roles, respectively, in phyllody induction.

**FIGURE 6:**
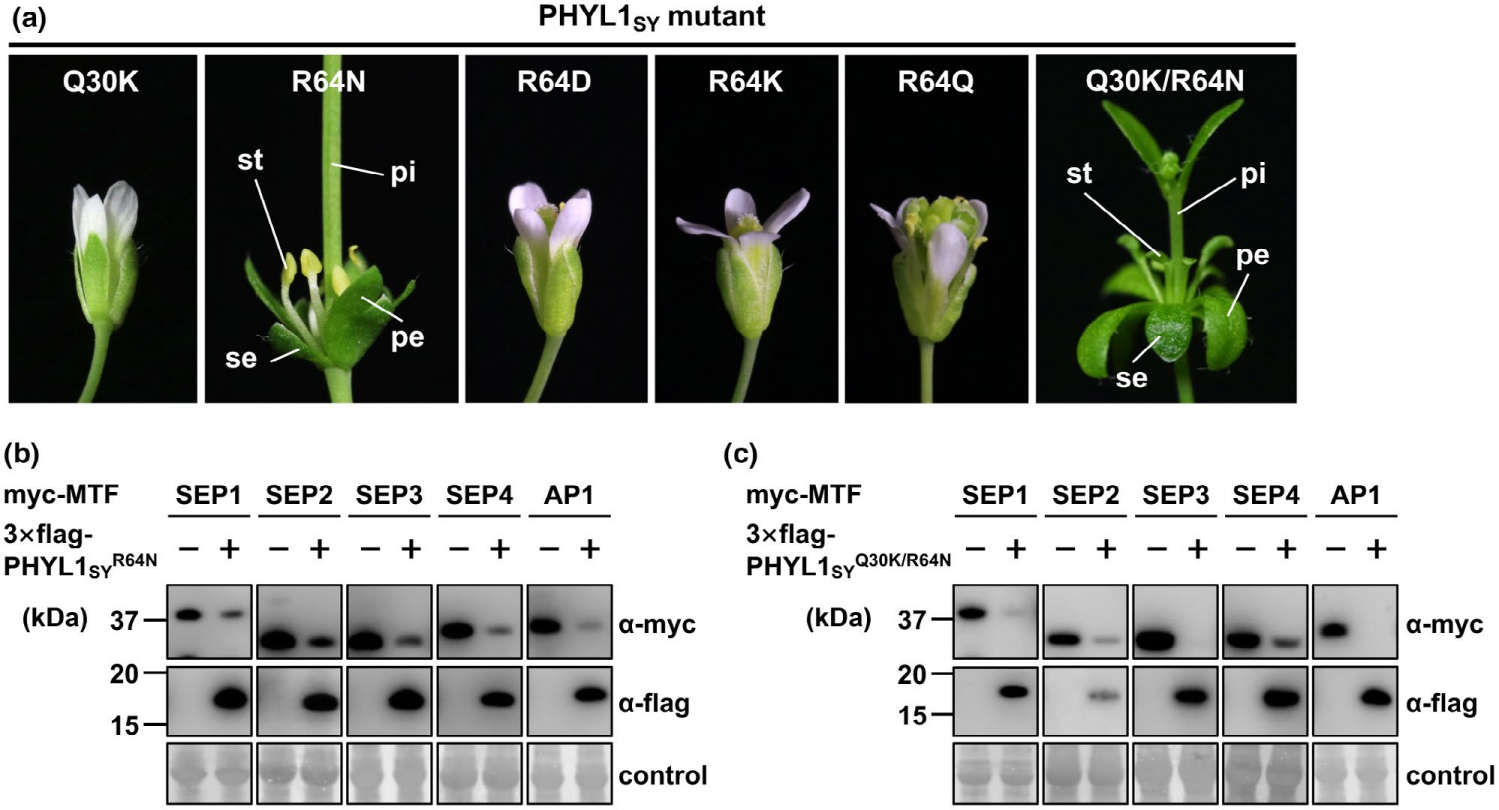
R64N substitution is sufficient for conferring phyllody‐inducing activity on PHYL1_SY_. (a) Floral phenotypes of *Arabidopsis thaliana* plants infected with tobacco rattle virus (TRV) vector carrying PHYL1_SY_ mutants at corresponding Q and R residues to K30 and N64 of PHYL1_OY_. Abbreviations of phyllody‐like phenotypes are described in Figure 3a. (b) and (c) Accumulation of transiently expressed Myc‐fused MADS domain transcription factors (MTFs; SEP1–4 and AP1) upon co‐expression with 3 × FLAG‐fused PHYL1_SY_^R64N^ (b) or 3 × FLAG‐fused PHYL1_SY_^Q30K/R64N^ (c). In planta protein expression and detection were performed as described in Figure 4

To test whether these residues are involved in the loss of phyllody‐inducing activity in the phyl‐B group, and the mechanisms if they are, we introduced reciprocal substitutions at positions 30 and 64 to those of the other groups into PHYL1_SY_. Although PHYL1_SY_^Q30K^ did not induce any flower malformation, PHYL1_SY_^R64N^ and PHYL1_SY_^Q30K/R64N^ induced similar phyllody phenotypes (Figure 6a), with slight differences in the morphology of stamens: PHYL1_SY_^Q30K/R64N^ induced more greenish and enlarged leaf‐like stamens compared to PHYL1_SY_^R64N^. In contrast, PHYL1_SY_ mutants at position 64 to other hydrophilic residues (asparagine acid [R64D], lysine [R64K], or glutamine [R64Q]) did not restore the phyllody‐inducing activity of PHYL1_SY_ (Figure 6a). Western blotting analyses showed that PHYL1_SY_^R64N^ decreased the amount of *Arabidopsis* AP1 and SEP1–4 MTFs compared to PHYL1_SY_ (Figure 6b) and that PHYL1_SY_^Q30K/R64N^ further enhanced activity (Figure 6c). These results indicate that N64R/K30Q substitutions also play critical and marginal roles, respectively, in the loss of phyllody‐inducing activity in the phyl‐B group. This supposition is supported by the fact that PHYL1_PWB_, which belongs to the phyl‐A group with Q30 (Figure 1a), also induced phyllody in *A*. *thaliana* (Figure 3a).

### 2.6 N64 residue regulates phyllogen–MTF interaction in cooperation with another polymorphic residue

**FIGURE 7:**
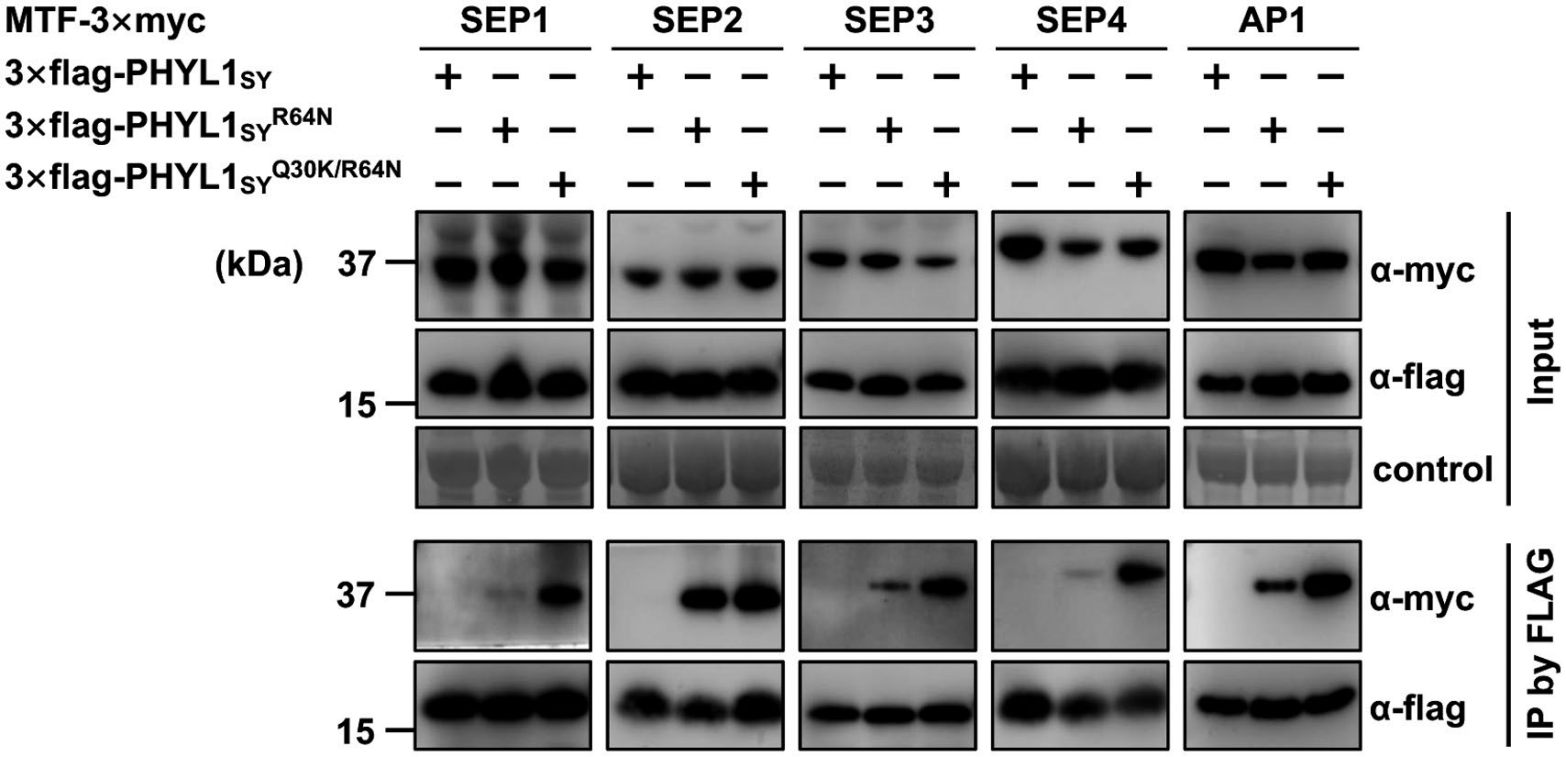
In planta interactions of PHYL1_SY_ mutants with floral MADS domain transcription factors (MTFs). *Agrobacterium* cultures expressing 3 × Myc‐fused SEP1–4 and AP1 and either 3 × FLAG fused PHYL1_SY_ or its mutants were mixed at a ratio of 1:1 and infiltrated into *Nicotiana benthamiana* leaves. Then, 36 hr after infiltration, total proteins were extracted and immunoprecipitation was performed with an α‐ FLAG antibody. The input and immunoprecipitated proteins (IP) were analysed by immunoblot analyses with α‐ FLAG and α‐Myc antibodies. Coomassie brilliant blue‐stained membranes are shown as a loading control

To gain further insight into the role of N64 residue in phyllody induction, we examined the interaction of PHYL1_SY_ mutants with host factors. Y2H assay showed that PHYL1_SY_^R64N^ enhanced interactions with SEP2, SEP3, SEP4, and AP1 compared to PHYL1_SY_ (Table 1 and Figure S10). PHYL1_SY_^Q30K/R64N^ interacted with the MTFs to the same extent as the phyllody‐inducing phyllogens. To examine in planta interactions with the A‐ and E‐class MTFs of PHYL1_SY_^R64N^ and PHYL1_SY_^Q30K/R64N^, we used co‐immunoprecipitation assays. SEP1‐, SEP2‐, SEP3‐, SEP4‐, and AP1–3 × Myc were coprecipitated with PHYL1_SY_^R64N^ and PHYL1_SY_^Q30K/R64N^ but not with PHYL1_SY_ (Figure 7). Furthermore, each MTF was more efficiently coprecipitated with PHYL1_SY_^Q30K/R64N^ compared to PHYL1_SY_^R64N^ (Figure 7). These results suggest that N64 residue regulates the interaction of phyllogens with these MTFs in cooperation with K30.

## 3 DISCUSSION

### 3.1 Biological implications of non‐phyllody‐inducing phyllogens

In this study, we identified new phyllogen genes of a wide variety of phytoplasmas (Table S1). The phyllogen family was composed of four distinct groups: phyl‐A, ‐B, ‐C, and ‐D (Figure 2). Phyllogens of phyl‐A, ‐C, and ‐D had phyllody‐inducing activity (Figure 3a), decreased the amount of SEP1–4 and AP1 in planta (Figure 4a,c,d; Maejima *et al*., 2014, 2015), and interacted with floral MTFs and RAD23C/D (Table 1), with the exception of α‐helix truncated phyllogens (Figures 3d, S3 and Table 1). In contrast, all members of the phyl‐B group failed to induce any flower malformation (Figures 3b,d and S7) or interact with MTFs while maintaining their interactions with SEP1 and RAD23C/D to the same extent as phyllody‐inducing phyllogens (Table 1). Furthermore, PHYL1_SY_, a representative of the phyl‐B group, did not decrease the amount of SEP1–4 and AP1 in planta (Figure 4b).

Although diverse phytoplasmas frequently induce phyllody symptoms (Chaturvedi *et al*., 2010; Musetti and Pagliari, 2019), there is little information on how functional diversity in effectors is involved in phytoplasma pathogenesis. In a variety of phyllogen families of diverse phytoplasmas, we identified phyllody‐inducing phyllogens from phyllody‐inducing phytoplasmas such as “*Ca*. P. japonicum” JHP and “*Ca*. P. aurantifolia” FBP (Figure 3a). In contrast, phyllody is not generally observed in diseased plants infected with phytoplasmas harbouring non‐phyllody‐inducing phyllogens, such as the phyl‐B group (Jung *et al*., 2003; Jiang *et al*., 2004; Tanaka *et al*., 2008). These results strongly suggest that natural functional variation in phyllogens is associated with the aetiology of phyllody in phytoplasma diseases. Considering that phytoplasmas harbouring the phyl‐B group derived from trees or vegetatively propagating plants (mulberry, porcelain vine, strawberry for PHYL1_MD_, PHYL1_PvWB_, and PHYL1_SY_, respectively), phyllody‐inducing activity could be dispensable for improvement of phytoplasma fitness in these perennial plants.

However, phyl‐B group phyllogens lacking the ability to induce phyllody still interacted with host factors, but the interaction specificity was different from that of phyllody‐inducing phyllogens—the former phyllogens interacted with RAD23C/D to the same extent as the latter phyllogens but not always with floral MTFs (Table 1). Reciprocal substitution analyses indicated that the phyl‐B‐group‐specific substitution of a polymorphic residue (R64) contributed to changes in interaction specificity with floral MTFs (Table 1 and Figure 7). These data may indicate that phyl‐B group phyllogens are not merely loss‐of‐function mutants but are a novel class of phyllogens that still have unresolved functions that are independent of phyllody induction. As an example of a phyllody‐independent function, SAP54, a phyllogen belonging to phyl‐A group that interacts with diverse non‐floral MTFs (MacLean *et al*., 2014), contributes to insect–vector attraction in a RAD23‐dependent but phyllody‐independent manner in *A*. *thaliana* (Orlovskis and Hogenhout, 2016). Although the mechanisms of the phyllody‐independent function of the phyllogen family are still unknown, further functional elucidation of the non‐phyllody‐inducing phyl‐B group may reveal unresolved roles of the effector family that improve phytoplasma fitness.

### 3.2 Phyllogen genes can be horizontally transferred among diverse phytoplasmas

To date, there is no universal and efficient method of identifying a variety of phyllogens from diverse phytoplasmas. To overcome this limitation, we developed a new approach based on PCR targeting of two conserved α‐helical regions of the protein family (Figure S2). Our identified phyllogens shared several conserved sequence characteristics with the well‐known phyllogens, such as the ribosome‐binding site, start and stop codons (Figure S3), signal peptide cleavage site, and two α‐helical secondary structures (Figure 1a), which indicates that each phyllogen is actually translated and secreted by the bacteria. As a result, this and previous studies have demonstrated that at least 59 strains related to 11 phytoplasma species (Table S1) have phyllogens with high sequence and evolutionary diversity (Table S4, Figures 2 and S5).

Although functional analyses of several effector homologs of phytoplasma have become areas of active research (Sugawara *et al*., 2013; Maejima *et al*., 2014; Chang *et al*., 2018; Wang *et al*., 2018b; Pecher *et al*., 2019), the evolutionary dynamics of these homologs have not yet been addressed. In the current study, phylogenetic analyses indicated that the phyllogen family evolved independently of “*Ca*. Phytoplasma” species, represented by the 16S rRNA phylogeny (Figures 2 and S5). Note that phyllogens found in closely related phytoplasmas (“*Ca*. P. asteris” and “*Ca*. P. pruni”) were separated into several different phylogenetic groups (Figure 2b). It is reasonable to hypothesize that phyllogens were transferred horizontally among phylogenetically distinct phytoplasmas. The acquisition of new genes via horizontal gene transfer contributes to the evolution of bacterial pathogens, particularly to the generation of new variants (Hacker *et al*., 1997; Pallen and Wren, 2007). Although the molecular mechanism of horizontal gene transfer among phytoplasmas remains poorly understood, PMUs are good candidates for the genetic mobile elements in diverse phytoplasma species because of their transposon‐like elements (Toruño *et al*., 2010; Chung *et al*., 2013; Ku *et al*., 2013). Most phyllogens belonging to phyl‐A are associated with PMUs (Jomantiene *et al*., 2007; Sugio and Hogenhout, 2012; Maejima *et al*., 2014). In one study, a phyllogen of the JWB‐nky strain (phyl‐D group) was located around a PMU (Wang *et al*., 2018a). These findings suggest that horizontal gene transfer of phyllogens by PMUs has contributed to the acquisition and sharing of phyllody‐inducing activity among phytoplasmas. Phytoplasmas have a wide plant host range and can naturally co‐infect the same host plants (Wei *et al*., 2016), whereas insect transmission is specific between different phytoplasmas and distinct insect vector taxa (Gonella *et al*., 2019). These results suggest that the co‐infection of phylogenetically diverse phytoplasma strains/species in their common plant hosts can facilitate horizontal gene transfer among phytoplasma genomes. Interestingly, however, it remains unclear how phyllogens originated due to the unavailability of potential ancestral protein sequences that are homologous with the phyllogen family, other than the phytoplasma genome, as previously mentioned (Rümpler *et al*., 2015). Further insights into the ancestral protein sequences of phyllogen genes are needed to make it possible to trace the comprehensive evolutionary trajectory of the phyllogen family during phytoplasma evolution.

### 3.3 Loss of phyllody‐inducing activity attributable to a polymorphic residue and α‐helix truncation

Mutational analyses of phyllogens revealed that the loss of phyllody‐inducing activity in the phyl‐B group was attributable to a polymorphic residue at position 64 (Figures 5b and 6a) that regulates the MTF‐binding and ‐degrading activity of the effector in cooperation with another polymorphic residue at position 30 (Figures 6b,c and 7 and Table 1). Thus, we identified a loss of phyllody‐inducing activity attributable to α‐helix truncation or a polymorphic residue. Several phytoplasma effectors (phyllogen, TENGU, and SAP11) induce the unique symptoms observed in phytoplasma‐infected plants (Namba, 2019). Although phyllogen and SAP11 homologs exhibit functional variation, including the ability to interact with and degrade their targets (Maejima *et al*., 2014; Chang *et al*., 2018; Pecher *et al*., 2019), it remains unclear how polymorphisms determine differences in effector function because of limited data on each effector family. Our finding of natural variation in the function of phytoplasma effectors attributable to a single amino acid provide the first molecular insights into polymorphisms in single effector‐mediated pathogenesis of phytoplasmas.

### 3.4 Contribution of N64 residue to α‐helix‐mediated interaction with MTFs of phyllogen

The phyllogen–MTF interaction is suggested to depend on hydrophobic interaction between the α‐helices based on two alanine insertions (Iwabuchi *et al*., 2019) or two amino acid substitutions (Liao *et al*., 2019) into hydrophobic residues within α‐helices of phyllogens. Further supporting this notion, we found that the two α‐helices with conserved hydrophobic residues were also found in the newly identified phyllogens (Figures 1 and S6), suggesting that two α‐helices are structural bases for the interactions of phyllogen with host factors as in the case of the K domain‐mediated tetramerization of MTFs (Puranik *et al*., 2014).

How then is the hydrophilic residue at position 64 involved in the putative hydrophobic interaction between phyllogens and MTFs? One hypothesis is that the N64 residue plays a key role in α‐helix formation and the N64R substitution disrupts the structural integrity; however, the interaction of the phyl‐B group with SEP1 and RAD23C/D in yeast cells (Table 1) indicates that N64R substitution does not disrupt the overall structure of phyllogens. Another hypothesis is that the phyllogen–MTF interaction is mediated by both hydrophilic and hydrophobic residues. Unlike conserved hydrophobic residues oriented toward the interiors of both α‐helices, N64 is exposed on the conserved surface of the phyllogen along with conserved external hydrophobic residues (Figures 1b and 5a). Particularly, two external hydrophobic residues in PHYL1_PnWB_ (L61 and L65 based on PHYL1_OY_), which also contribute to the coordinated interaction with SEP3 (Liao *et al*., 2019), were exposed on the same side as and adjacent to the N64 residue (Figure S9). For SEP3 oligomerization, hydrophilic residues flanking hydrophobic residues could play a significant role in the stabilization of α‐helix‐mediated protein interaction (Puranik *et al*., 2014). These results suggest that the external surface structures of phyllogens formed by these hydrophobic and hydrophilic residues are important for phyllogen–MTF interactions. In addition, K30 exposed on the same side of N64 (Figures 1b, 5a and S9) can also contribute marginally to the interaction in a similar manner. The amino acid substitutions at position 64 showed that even glutamine, whose side chain has similar properties to asparagine, was not sufficient to confer phyllody‐inducing activity on PHYL1_SY_ (Figure 6a). Because glutamine generates more steric hindrance due to its larger size compared to asparagine (Bogan and Thorn, 1998), the steric property of N64 may be crucial for the stabilization of phyllogen–MTF interaction. These results suggest that while the two α‐helices are the structural bases for the interaction with host factors, phyllogens have acquired functional variation via their amino acid polymorphisms on α‐helices, which may improve the fitness of each phytoplasma. Comprehensive interaction analyses among each phyllogen focusing on its polymorphisms will elucidate the structural mechanism underlying phyllody induction and contribute to controlling phyllody symptoms caused by phytoplasma.

## 4 EXPERIMENTAL PROCEDURES

### 4.1 DNA samples

Table S1 lists the DNA samples of the phytoplasmas used in this study with the full strain names and GenBank accession numbers. DNA samples of the FBP, SY, and PvWB strains were amplified before use for whole‐genome amplification using the REPLI‐g Mini Kit (Qiagen) according to the manufacturer's instructions. DNA samples of PoiBI and ChV strains were extracted from a PoiBI‐infected commercial poinsettia cultivar and ChV‐infected periwinkle, respectively, with the QIAamp DNA Mini Kit (Qiagen).

### 4.2 PCR analyses

Table S2 lists the primers used in this study. A degenerate primer pair, PHYL‐F/R (Figure S2a), was designed for two conserved regions in three phyllogen gene sequences (*PHYL1_OY_*, *PHYL1_PnWB_*, and *SAP54*). PCR was performed in 10 µl reaction volumes containing KOD FX (Toyobo) master mix, with 0.3 µM of each primer, and 10 ng of genomic DNA from each phytoplasma‐infected plant. The PCR conditions consisted of an initial denaturation at 94°C for 3 min; 35–45 cycles of 94°C for 15 s, 45°C for 30 s, and 68°C for 30 s; with a final 7 min extension at 68°C. 16S rRNA gene sequences of several phytoplasmas were determined using the methodology described in Iwabuchi *et al*. (2018). Phyllogens of MD, SWB, PWB, and PoiBI strains were identified in each genomic DNA sample using primers based on up‐ and downstream sequences of the known phyllogens indicated as “Target gene” in Table S2. The PCR amplicons were purified followed by direct sequencing with a BigDye Terminator kit (Applied Biosystems) and each of the primers used in PCR.

### 4.3 Genome walking

Up‐ and downstream sequences of the partially amplified phyllogens were determined with genome walking using APAgene Gold Genome Walking kit (Bio S&T) according to the manufacturer's instructions. A total of 100 ng of either genomic DNA or whole‐genome‐amplified DNA was used as a template. The purified PCR amplicons were TA‐cloned into pCR4‐TOPO vector (Invitrogen) and sequenced with gene‐specific primers (GSPs), M13F, and M13R primers. The sequence information obtained, a partial phyllogen gene sequence and its up‐ and downstream sequences, was assembled with ATGC v. 4.3.5 (GENETYX). To validate the assembled sequences, PCR analyses were performed using primers designed on the up‐ and downstream sequences of each phyllogen (Figure S2b).

### 4.4 Identification of ChV strain phyllogens using MiSeq sequencing

Phyllogens of the ChV strain were identified with draft‐genome sequencing using an Illumina MiSeq sequencer. A library was prepared with a Nextera XT DNA Sample Prep Kit (Illumina), according to the manufacturer's instructions. It was run on a MiSeq sequencer that provided paired reads 150 nt long. The CLC Genomic Workbench (CLC‐bio) was used to map the phytoplasma draft‐genome sequence of PnWB phytoplasma (GenBank assembly accession GCA_000364425.1). Two phyllogens of the ChV strain, *PHYL1_ChV_* and *PHYL1_ChV_^Ψ^*, were identified with a homology search with tblastn (Camacho *et al*., 2009). An approximately 1.6 kb genomic region containing *PHYL1_ChV_* and an approximately 0.9 kb genomic region containing *PHYL1_ChV_^Ψ^* were amplified by PCR with primers AUR‐1/AUR‐2 and AUR‐3/AUR‐4, respectively. These PCR products were sequenced as described previously.

### 4.5 Phyllogen sequence analyses and structural modelling

The nucleotide and amino acid sequences of the phyllogen family were aligned with the MUSCLE algorithm (Edgar, 2004). Consensus secondary structure elements were predicted with PROMALS3D (Pei *et al*., 2008). The web‐based SignalP v. 4.1 server (http://www.cbs.dtu.dk/services/SignalP/) was used to analyse the presence and location of signal peptide cleavage sites. The sequence identities were calculated with the Sequence Demarcation Tool (SDT) v. 1.2 (Muhire *et al*., 2014). Phylogenetic trees based on partial or complete nucleotide sequences of the phyllogen family and 16S rRNA gene sequences were constructed with MEGA v. 7.0 using the neighbour‐joining method, as detailed in Iwabuchi *et al*. (2018). Two identical copies of phyllogen at two locations in complete genome sequence of “*Ca*. P. ziziphi” JWB‐nky strain (Wang *et al*., 2018a) were treated as a single sequence. Structures were modelled with the I‐TASSER server (Zhang, 2008) and selected based on the C‐score, which is a confidence score used to estimate the quality of the predicted models. The C‐score is typically in the range of [−5,2], where a C‐score of higher value signifies a model with a high confidence and vice versa. Using a C‐score cutoff >−1.5 for the models of correct topology, both false positive and false negative rates are below 0.1 (Zhang, 2008). Structure visualization and hydrophobicity calculation based on the Kyte and Doolittle scale were performed with UCSF Chimera (Pettersen *et al*., 2004). Sequence conservation scores were calculated using the ConSurf server (Ashkenazy *et al*., 2016).

### 4.6 Cloning and site‐directed mutagenesis of phyllogens

The predicted coding regions of the phyllogens, excluding their signal peptides, were cloned. *PHYL1_PYR_* and plant codon‐optimized *PHYL1_OY_* and *PHYL1_PnWB_* were previously cloned into the pENTA vector (Maejima *et al*., 2014; Kitazawa *et al*., 2017; Iwabuchi *et al*., 2019). Seven other phyllogens were plant codon‐optimized and synthesized by Thermo Fisher Scientific (Figure S1). *PHYL1_231/09_*, *PHYL1_JHP_*, *PHYL1_JWB_*, *PHYL1_SY_*, and *PHYL1_PWB_* were cloned into the pENTA vector using the methodology described by Kitazawa *et al*. (2017). *PHYL1_FBP_* and *PHYL1_PvWB_* were cloned into the pTRV2 vector with additional sequences cloned into the pTRV2 vector (5′‐TCCAACCCTGGGCCC‐3′ at their 5′ end and 5′‐TAGGGATTTAAGGAC‐3′ at their 3′ end). Prior to cloning into the pENTA vector, the *PHYL1_FBP_* and *PHYL1_PvWB_* fragments were amplified from those synthesized genes. *PHYL1_MD_* was generated by adding site‐directed mutations into pENTA‐cloned *PHYL1_PvWB_* using the GeneArt site‐directed mutagenesis system (Invitrogen). Amino acid substitution mutants of phyllogens were generated in the same way.

### 4.7 Yeast two‐hybrid analyses

The Matchmaker GAL4 Two‐Hybrid System 3 kit (Clontech) was used. Activation domain (AD)‐fused AP1 and SEP1, SEP2 (aa 87–220), SEP3, and SEP4 (aa 57–257), RAD23C and BD‐fused PHYL1_OY_, PHYL1_PnWB_, and PHYL1_PYR_ were constructed previously (Maejima *et al*., 2014; Kitazawa *et al*., 2017; Iwabuchi *et al*., 2019). To construct AD‐fused RAD23D, the *RAD23D* gene of *A*. *thaliana* (NM_001085211) was cloned into the pGADT7 vector (Clontech) using *Nde*I and *Eco*RI sites. To construct DNA‐binding domain (BD)‐fused PHYL1_231/09_, PHYL1_FBP_, PHYL1_JWB_, PHYL1_MD_, PHYL1_PvWB_, PHYL1_PWB_, PHYL1_PnWB_, and PHYL1_SY_ and their mutants, these fragments were cloned into the pGBKT7 vector (Clontech) using the same restriction sites. Co‐transformation of yeast cells and evaluation of protein interaction were performed as described by Iwabuchi *et al*. (2019).

### 4.8 In planta protein expression and detection

*A*. *thaliana* was maintained in a growth chamber with 16 hr light/8 hr dark conditions at 22°C. *N. benthamiana* was grown under natural light conditions at 25°C. For transient in planta expression of Myc‐fused SEP1, SEP2, SEP4, and AP1, triple c‐Myc (3 × Myc)‐fused SEP1–4 and AP1, and triple FLAG (3 × FLAG)‐fused PHYL1_231/09_, PHYL1_OY_, PHYL1_SY_, PHYL1_PnWB_, and their mutants, these genes were subcloned into pEarleyGate203 (Earley *et al*., 2006), pEarleyGateC3myc (Okano *et al*., 2014), and pEarleyGateN3 × FLAG (Iwabuchi *et al*., 2019), respectively, using Gateway LR Clonase II enzyme mix (Invitrogen). Myc‐fused SEP3 was constructed previously (Iwabuchi *et al*., 2019). *Agrobacterium*‐mediated transient expression and protein detection were performed using the methodology described by Iwabuchi *et al*. (2019).

A modified TRV‐based gene expression vector system (Iwabuchi *et al*., 2019) was used for stable in planta gene expression of phyllogens and their mutants. *Agrobacterium tumefaciens* EHA105 cells containing pTRV1 and each of the pTRV2‐containing CP fused with each of the phyllogen genes via the FMDV 2A peptide were adjusted to an OD_600_ of 1.0, mixed at a ratio of 1:1, and co‐infiltrated into 3‐ or 4‐week‐old *A*. *thaliana* and 4‐ or 5‐week‐old *N*. *benthamiana* leaves. Virus infection and foreign gene retention were confirmed by RT‐PCR using the methodology described in Iwabuchi *et al*. (2019).

### 4.9 Transgenic plants

*PHYL1_MD_*, *PHYL1_PvWB_*, *PHYL1_SY_*, and *PHYL1_PYR_* were subcloned from pENTA into the binary plasmid vector pFAST‐G02 (Inplanta Innovations) under the control of the cauliflower mosiac virus 35S promoter using Gateway LR Clonase II enzyme mix (Invitrogen). *A*. *tumefaciens* was transformed with each of the constructs, and *A*. *thaliana* plants were transformed using the floral‐dip method using the methodology described in Hoshi *et al*. (2009). T_1_ seeds of the transgenic plants were selected under fluorescence stereomicroscopy. The expression of each phyllogen was confirmed by RT‐PCR.

### 4.10 In planta immunoprecipitation

*Agrobacterium* cultures expressing 3 × Myc‐fused SEP1–4, AP1, and either 3 × FLAG‐PHYL1_SY_ or its mutants were mixed at a ratio of 1:1 and infiltrated into *N*. *benthamiana* leaves. The inoculated leaves were homogenized (500 mg/ml) in 1 × RIPA buffer (Yamaji *et al*., 2006) containing complete mini EDTA‐free protease inhibitors (Roche) and 0.1% 3‐mercapto‐1,2‐propanediol 36 hr after infiltration. The homogenate was centrifuged at 18,000 × g for 30 min at 4°C to remove debris. Then 60 µl of the supernatant was heat‐denatured in SDS sample buffer (Inputs). A total of 700–800 µl of the remaining supernatant was incubated for 90 min at 4°C with 30 µl of EZview Red ANTI‐FLAG M2 Affinity Gel (50% slurry in 1 × RIPA buffer; Sigma‐Aldrich). Following five washes with 800 µl of 1 × RIPA buffer, proteins bound to the beads were eluted with 30 µl of 1 × RIPA buffer containing 400 µg/ml 3 × FLAG peptide (Sigma‐Aldrich).

## Supporting information

Figure S1

Figure S2

Figure S3

Figure S4

Figure S5

Figure S6

Figure S7

Figure S8

Figure S9

Figure S10

Table S1

Table S2

Table S3

Table S4

## Data Availability

The data supporting these findings are available in DDBJ/EMBL/GenBank at https://www.ncbi.nlm.nih.gov/ under the accession numbers listed in Table S1.
