# Supplementary material for: Functional variation in phyllogen, a phyllody‐inducing phytoplasma effector family, attributable to a single amino acid polymorphism
Source: Mol Plant Pathol. 2020 Aug 19;21(10):1322–36. doi: 10.1111/mpp.12981 (PMC7488466; doi:10.1111/mpp.12981)
Supplement: Supplementary file 1 — Figure S1 [file MPP-21-1322-s001.pdf]

Figure S1

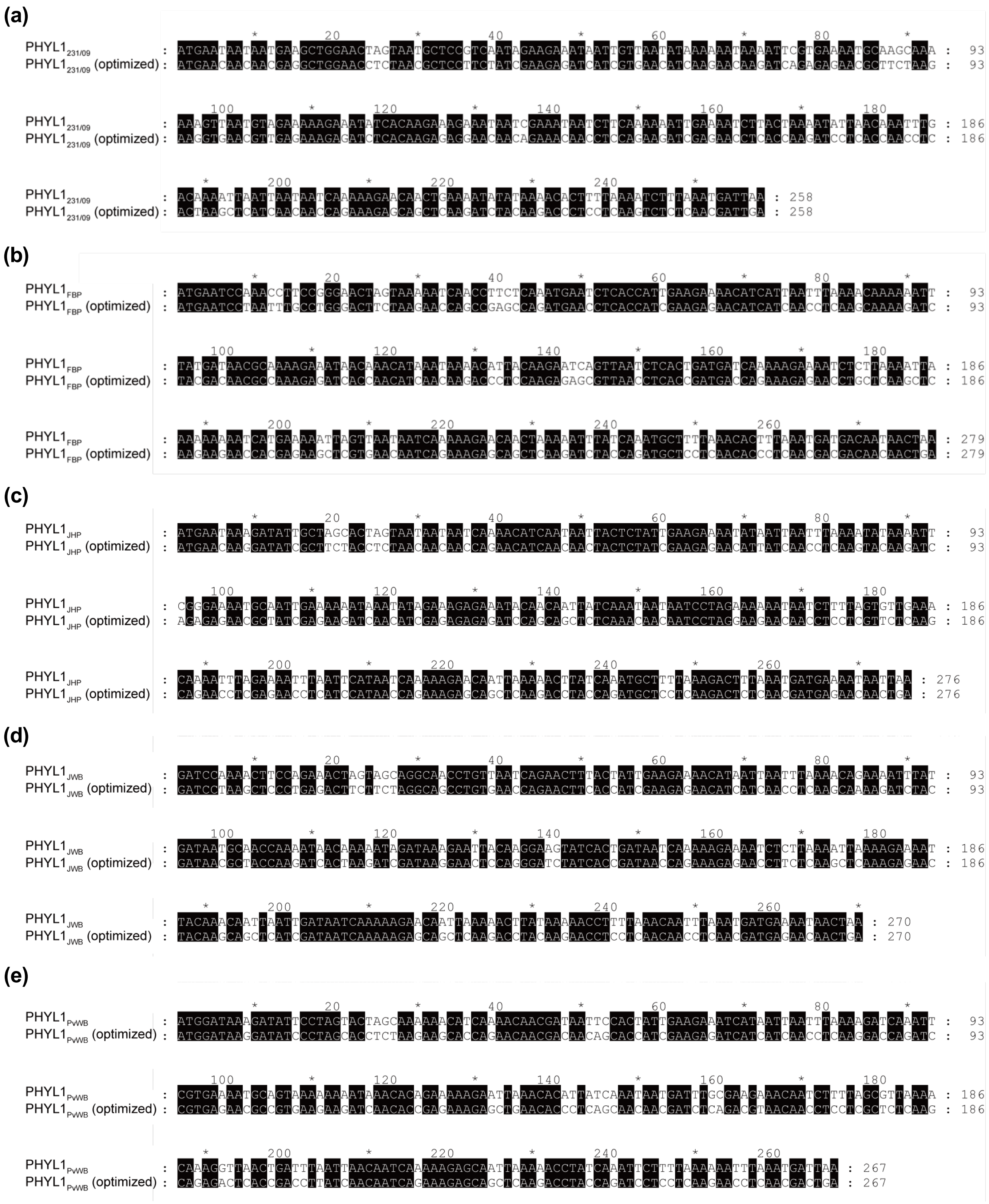

(a)
PHYL1231/09
PHYL1231/09 (optimized)
(b)
PHYL1FBP
PHYL1FBP (optimized)
(c)
PHYL1JHP
PHYL1JHP (optimized)
(d)
PHYL1JWB
PHYL1JWB (optimized)
(e)
PHYL1PvWB
PHYL1PvWB (optimized)

Figure S1 continued

(f)

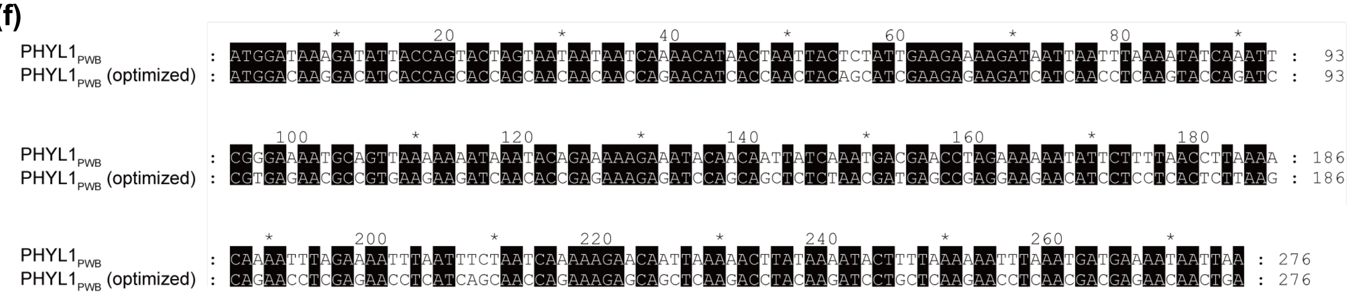

(g)

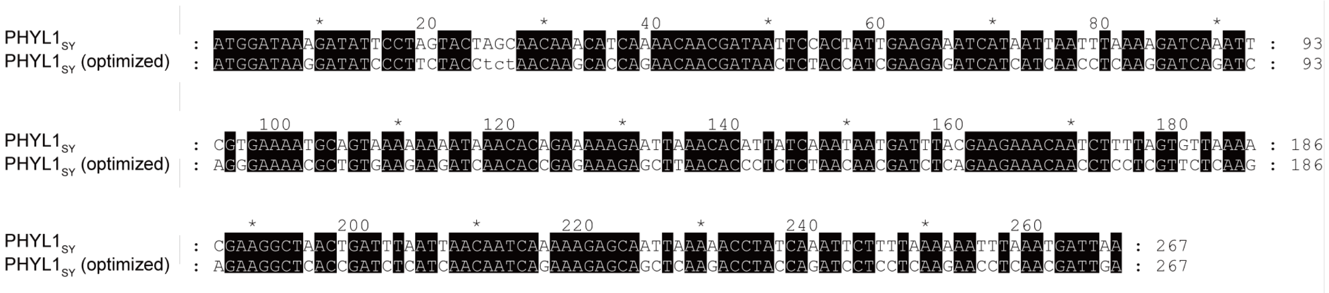

Figure S1. Plant-optimized nucleotide sequences of phyllogens used in this study. Plant-optimized nucleotide sequences of $PHYL1_{231/09}$ **(a)**, $PHYL1_{FBP}$ **(b)**, $PHYL1_{JHP}$ **(c)**, $PHYL1_{JWB}$ **(d)**, $PHYL1_{PvWB}$ **(e)**, $PHYL1_{PWB}$ **(f)**, and $PHYL1_{SY}$ **(g)**.
Conserved nucleotides are indicated by black boxes.
