# Supplementary material for: Functional variation in phyllogen, a phyllody‐inducing phytoplasma effector family, attributable to a single amino acid polymorphism
Source: Mol Plant Pathol. 2020 Aug 19;21(10):1322–36. doi: 10.1111/mpp.12981 (PMC7488466; doi:10.1111/mpp.12981)
Supplement: Supplementary file 2 — Figure S2 [file MPP-21-1322-s002.pdf]

Figure S2

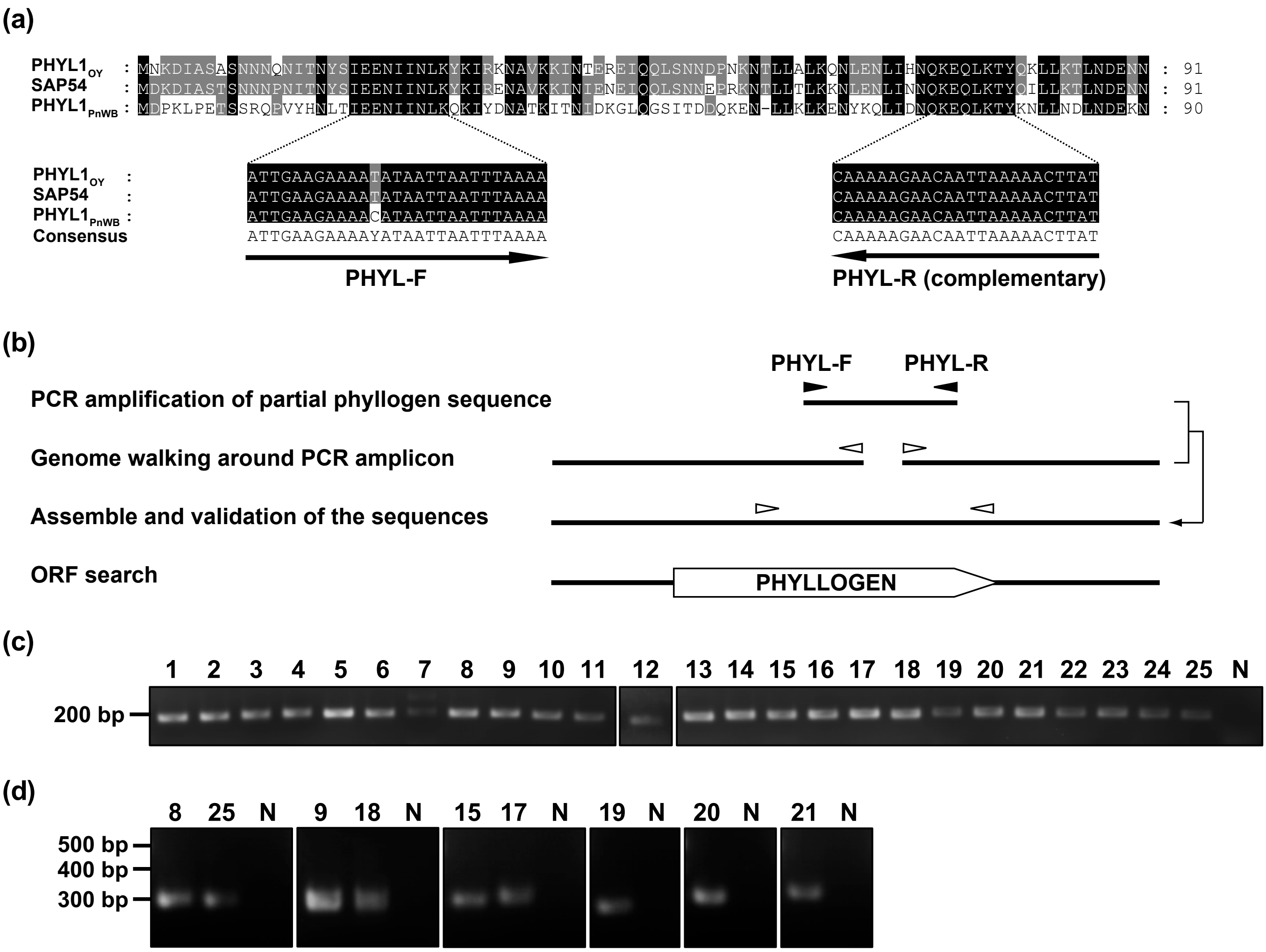

Figure S2. Identification of phyllogens based on conserved α-helical motifs.

(a) Multiple sequence alignment of three phyllogens ($PHYL1_{OY}$, $PHYL1_{PnWB}$, and SAP54). Dark gray and black shading indicate more than 60% and 100% consensus in each column, respectively. Black horizontal arrows show locations of the PHYL-F/R primers for amplification of the partial sequence of the phyllogen gene.

(b) Diagram for identifying phyllogens in the genomic DNA of phytoplasmas. Filled arrowheads indicate PHYL-F/R primers. Open arrowheads indicate gene-specific primers (GSPs) for each phyllogen.

(c) PCR amplification with PHYL-F/R of diverse phytoplasmas.

PCR analyses with the PHYL-F/R primer pair for detecting partial phyllogen genes of 25 phytoplasma strains. 1: AY-J, 2: CA, 3:KVF, 4: DIV, 5:GY, 6: HBWB, 7: HYDF, 8: HP, 9: PvWB, 10: RhY, 11: SWB, 12: MD, 13: CLP, 14: CrP, 15: FBP, 16: SOYP, 17: WBDL, 18: SY, 19: ASHy2, 20: JHP, 21: RYD, 22: NaxY, 23: LUM, 24: PWB, 25: JWB, N: distilled water (negative control). Full strain names and GenBank accession numbers are listed in Table S1.

(d) Validation of genome walking analysis.

PCR analyses with gene specific primers designed on up- and downstream sequences. Each phytoplasma strain names are shown as **(c)**.
