# Supplementary material for: Functional variation in phyllogen, a phyllody‐inducing phytoplasma effector family, attributable to a single amino acid polymorphism
Source: Mol Plant Pathol. 2020 Aug 19;21(10):1322–36. doi: 10.1111/mpp.12981 (PMC7488466; doi:10.1111/mpp.12981)
Supplement: Supplementary file 3 — Figure S3 [file MPP-21-1322-s003.pdf]

Figure S3

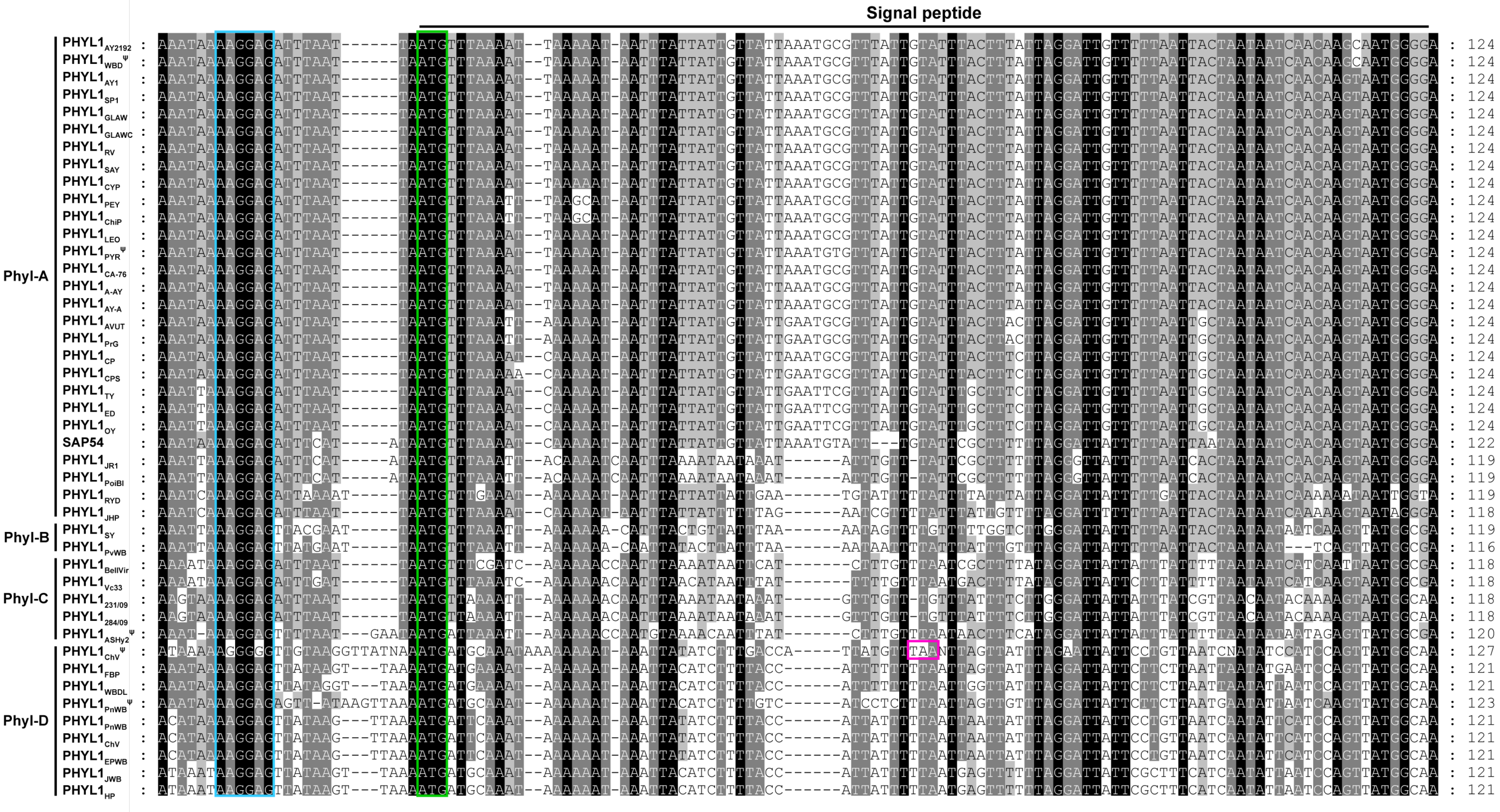
Signal peptide
Phyl-A
Phyl-B
Phyl-C
Phyl-D
PHYL1 AY2192
PHYL1 WBD Ψ
PHYL1 AY1
PHYL1 SP1
PHYL1 GLAW
PHYL1 GLAWC
PHYL1 RV
PHYL1 SAY
PHYL1 CYP
PHYL1 PEY
PHYL1 ChiP
PHYL1 LEO
PHYL1 PYR Ψ
PHYL1 CA-76
PHYL1 A-AY
PHYL1 AY-A
PHYL1 AVUT
PHYL1 PrG
PHYL1 CP
PHYL1 CPS
PHYL1 TY
PHYL1 ED
PHYL1 OY
SAP54
PHYL1 JR1
PHYL1 PoiBI
PHYL1 RYD
PHYL1 JHP
PHYL1 SY
PHYL1 PvWB
PHYL1 BellVir
PHYL1 Vc33
PHYL1 231/09
PHYL1 284/09
PHYL1 ASHy2 Ψ
PHYL1 ChV Ψ
PHYL1 FBP
PHYL1 WBDL
PHYL1 PnWB Ψ
PHYL1 PnWB
PHYL1 ChV
PHYL1 EPWB
PHYL1 JWB
PHYL1 HP

Figure S3 continued

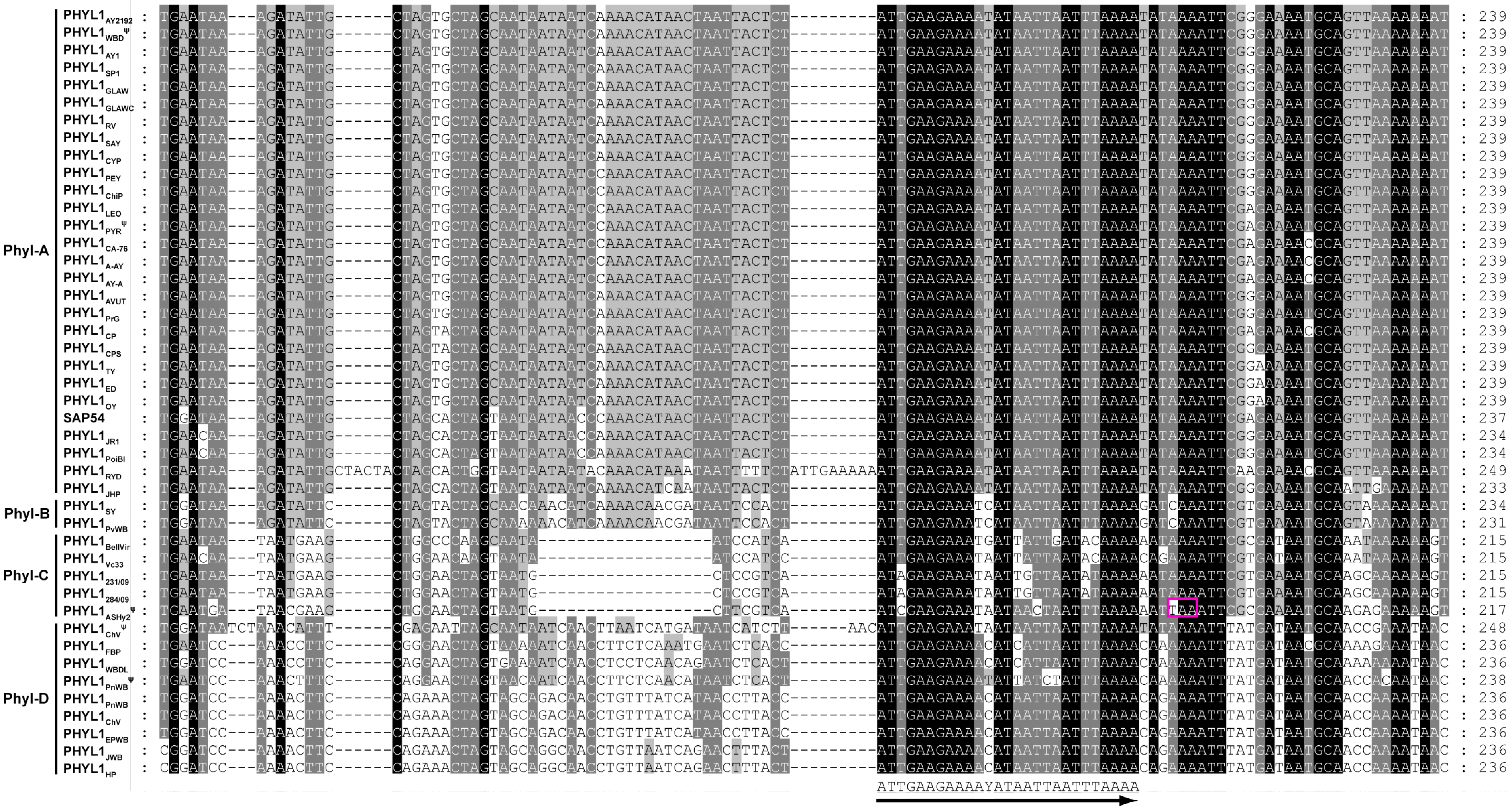

Figure S3 continued

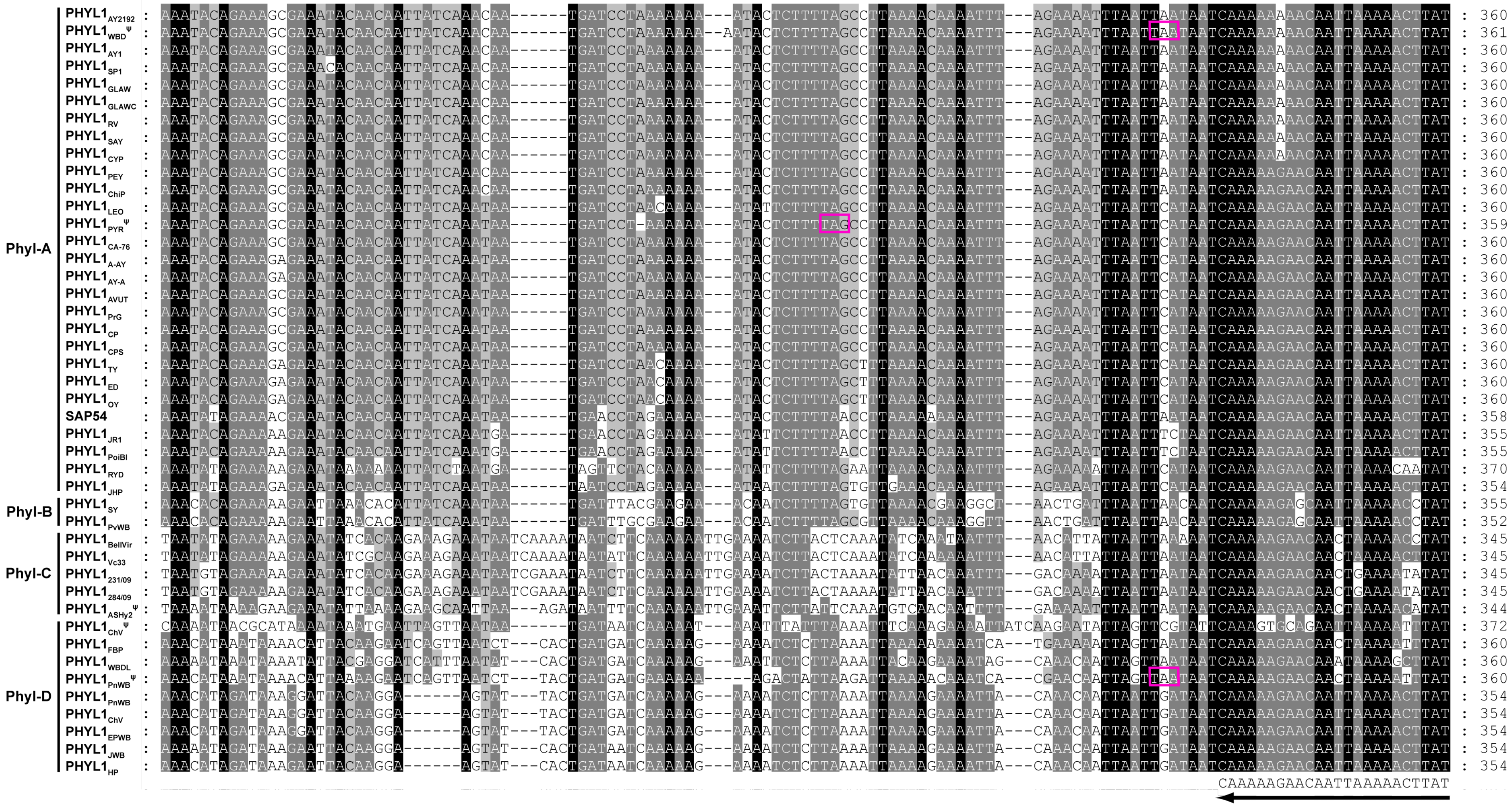

Figure S3 continued

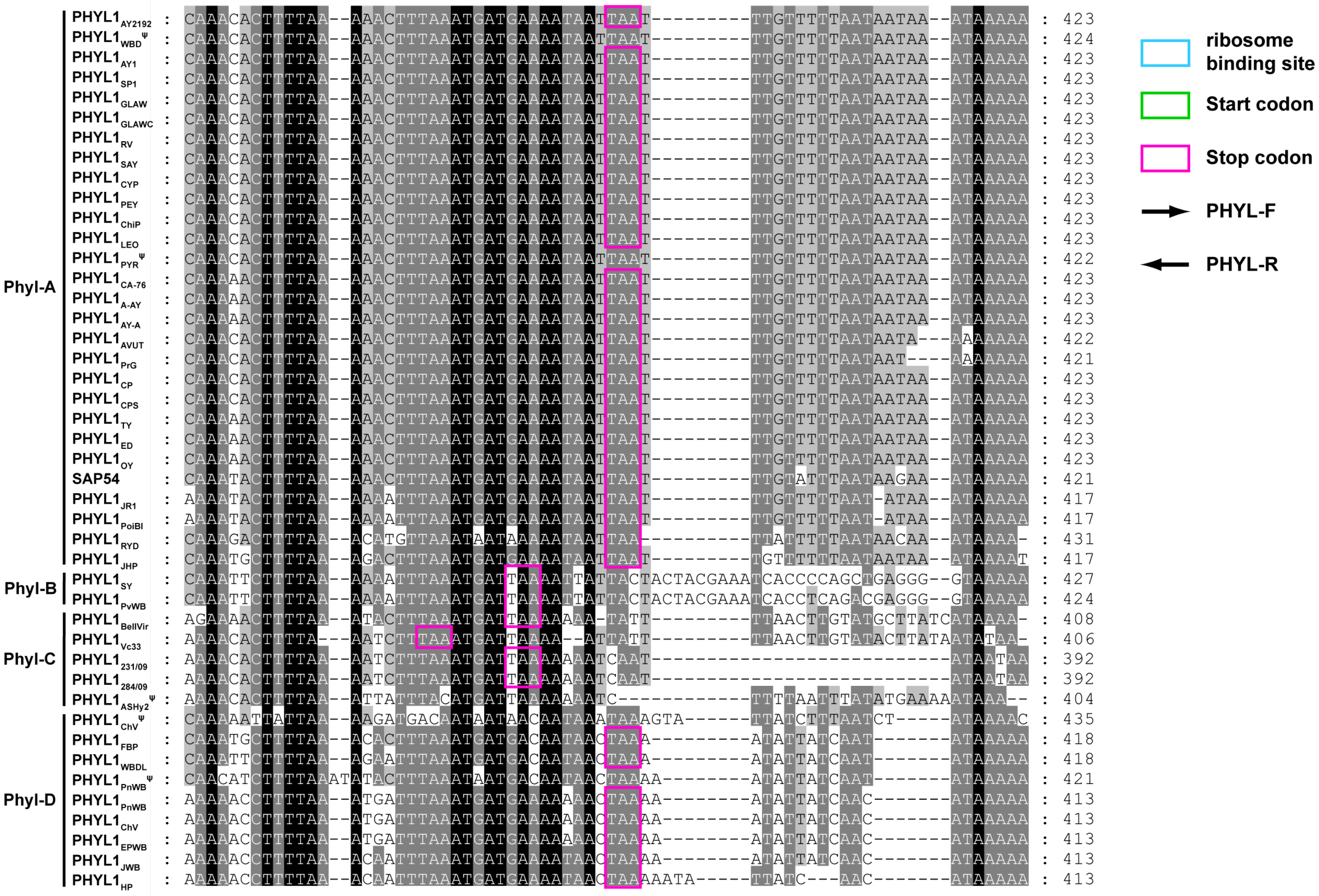

Figure S3. Alignment of full-length nucleotide sequences of the phyllogen family.
Nucleotide sequences of phyllogens with their up- and downstream regions were aligned. Light gray, dark gray, and black shading indicate more than 60%, more than 80%, and 100% consensus in each column, respectively. Ψ indicates a C-terminus truncated mutant due to a premature stop codon.
