# Supplementary material for: Functional variation in phyllogen, a phyllody‐inducing phytoplasma effector family, attributable to a single amino acid polymorphism
Source: Mol Plant Pathol. 2020 Aug 19;21(10):1322–36. doi: 10.1111/mpp.12981 (PMC7488466; doi:10.1111/mpp.12981)
Supplement: Supplementary file 4 — Figure S4 [file MPP-21-1322-s004.pdf]

Figure S4

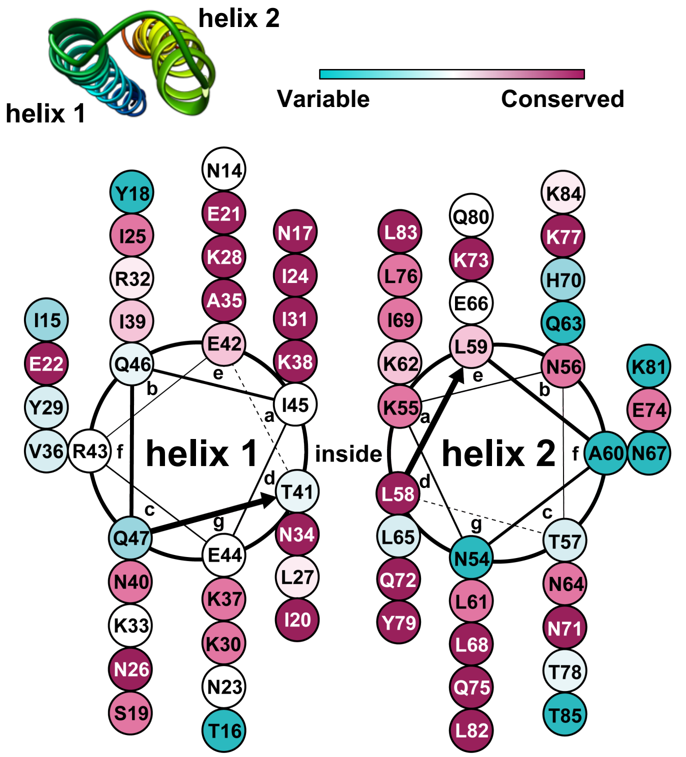

Figure S4. Sequence conservation scores shown on the helical wheel projection of PHYL1$_{OY}$. The helical wheel projection viewed from the C- and N-termini of helices 1 and 2, respectively, as indicated in the upper left. The number in each circle indicates an amino acid residue of PHYL1$_{OY}$. Heptad repeat positions are labeled a–g as described in Figure 1a. Colors in the circles represent sequence conservation scores based on Figure 1a. Conservation scores range from cyan (not conserved) to white (average) and to magenta (highly conserved).
