# Supplementary figures and images for: Functional variation in phyllogen, a phyllody‐inducing phytoplasma effector family, attributable to a single amino acid polymorphism
Source: Mol Plant Pathol. 2020 Aug 19;21(10):1322–36. doi: 10.1111/mpp.12981 (PMC7488466; doi:10.1111/mpp.12981)

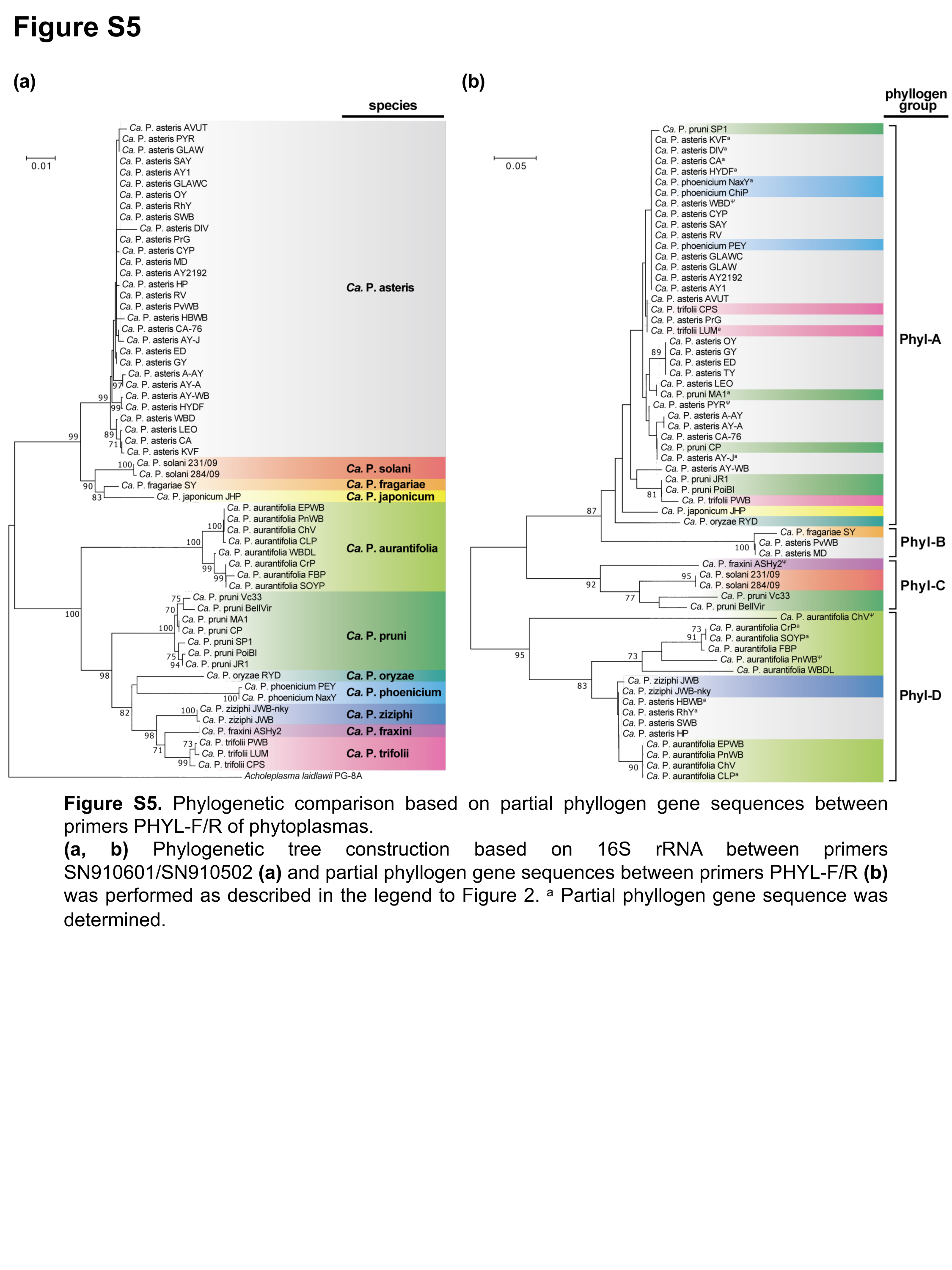

Supplement: Supplementary file 5 — Figure S5 [file MPP-21-1322-s005.jpg]
