# Supplementary material for: Functional variation in phyllogen, a phyllody‐inducing phytoplasma effector family, attributable to a single amino acid polymorphism
Source: Mol Plant Pathol. 2020 Aug 19;21(10):1322–36. doi: 10.1111/mpp.12981 (PMC7488466; doi:10.1111/mpp.12981)
Supplement: Supplementary file 6 — Figure S6 [file MPP-21-1322-s006.pdf]

Figure S6

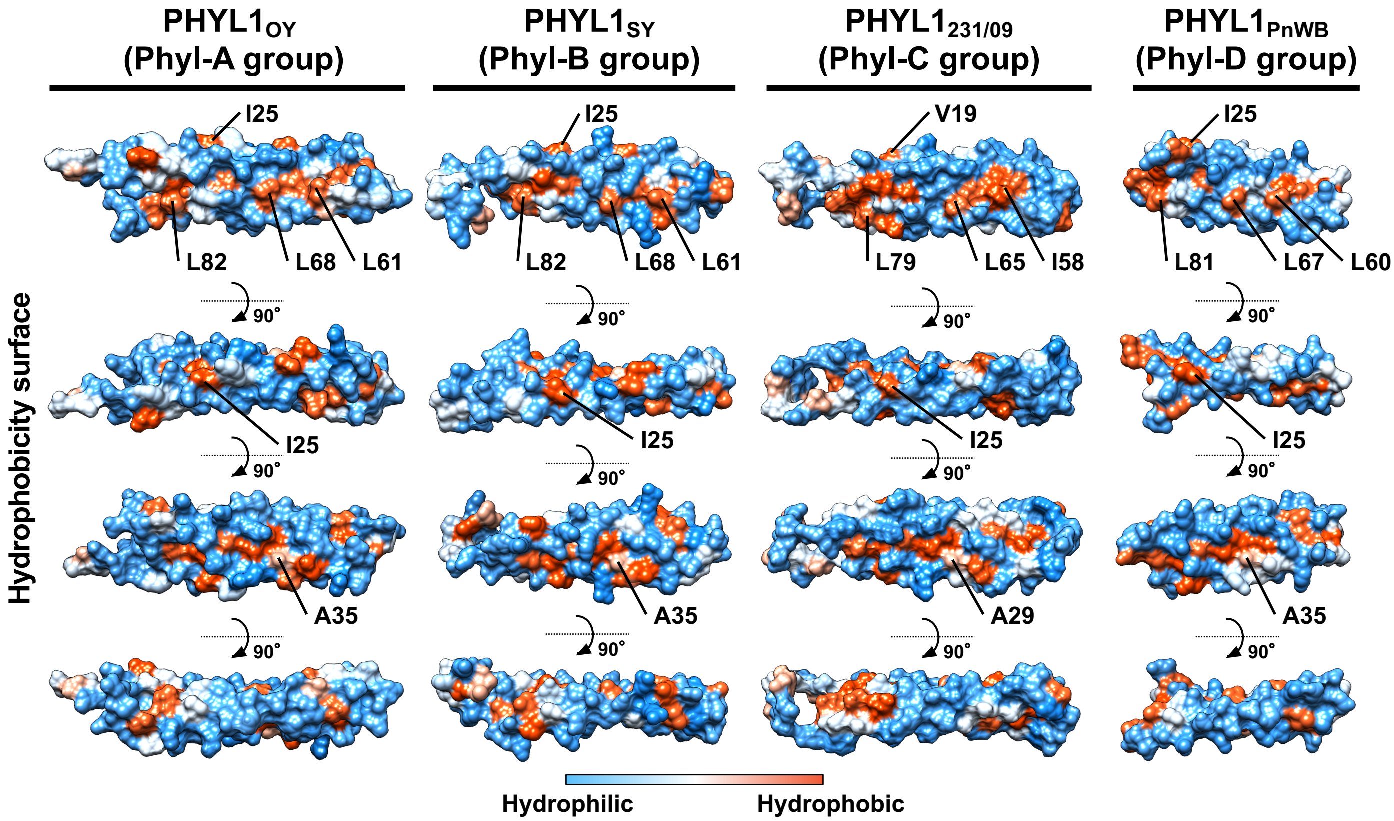

Figure S6. Structural comparison of the phyllogen family.
Structural modeling of PHYL1$_{SY}$ and PHYL1$_{231/09}$ belonging to the phyl-B and -C groups was performed based on the PHYL1$_{OY}$ (Phyl-A group) structure (residues 7–91 of subunit A without iodine atoms, PDB ID: 6JQA) using I-TASSER. Crystal structure of phyl-D group was based on the PHYL1$_{PnWB}$ (residues 19–84 of subunit B, PDB ID: 6INR). Hydrophobicity is calculated based on the Kyte and Doolittle scale in UCSF Chimera. Hydrophobicity scores range from blue (most hydrophilic) to white (average) and to orange red (most hydrophobic).
