# Supplementary material for: Functional variation in phyllogen, a phyllody‐inducing phytoplasma effector family, attributable to a single amino acid polymorphism
Source: Mol Plant Pathol. 2020 Aug 19;21(10):1322–36. doi: 10.1111/mpp.12981 (PMC7488466; doi:10.1111/mpp.12981)
Supplement: Supplementary file 7 — Figure S7 [file MPP-21-1322-s007.pdf]

Figure S7

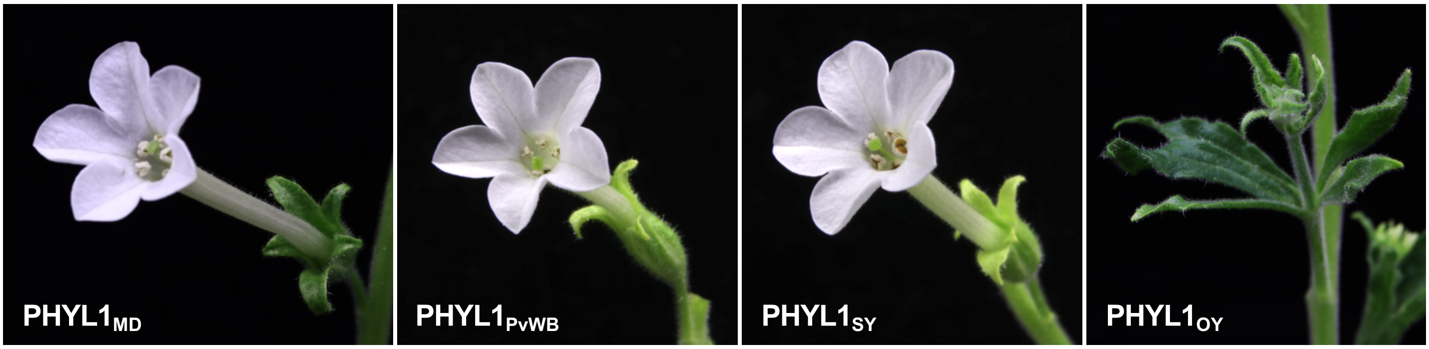

Figure S7. Floral phenotypes of *N. benthamiana* plants infected with TRV carrying phyllogens. Floral phenotypes of *N. benthamiana* plants infected with TRV vector carrying phyllogens belonging to either phyl-A ($PHYL1_{OY}$) or phyl-B ($PHYL1_{MD}$, $PHYL1_{PvWB}$, and $PHYL1_{SY}$) group. Phyllogens belonging to phyl-B ($PHYL1_{MD}$, $PHYL1_{PvWB}$, and $PHYL1_{SY}$) induced no flower malformation.
