# Supplementary material for: Functional variation in phyllogen, a phyllody‐inducing phytoplasma effector family, attributable to a single amino acid polymorphism
Source: Mol Plant Pathol. 2020 Aug 19;21(10):1322–36. doi: 10.1111/mpp.12981 (PMC7488466; doi:10.1111/mpp.12981)
Supplement: Supplementary file 8 — Figure S8 [file MPP-21-1322-s008.pdf]

Figure S8

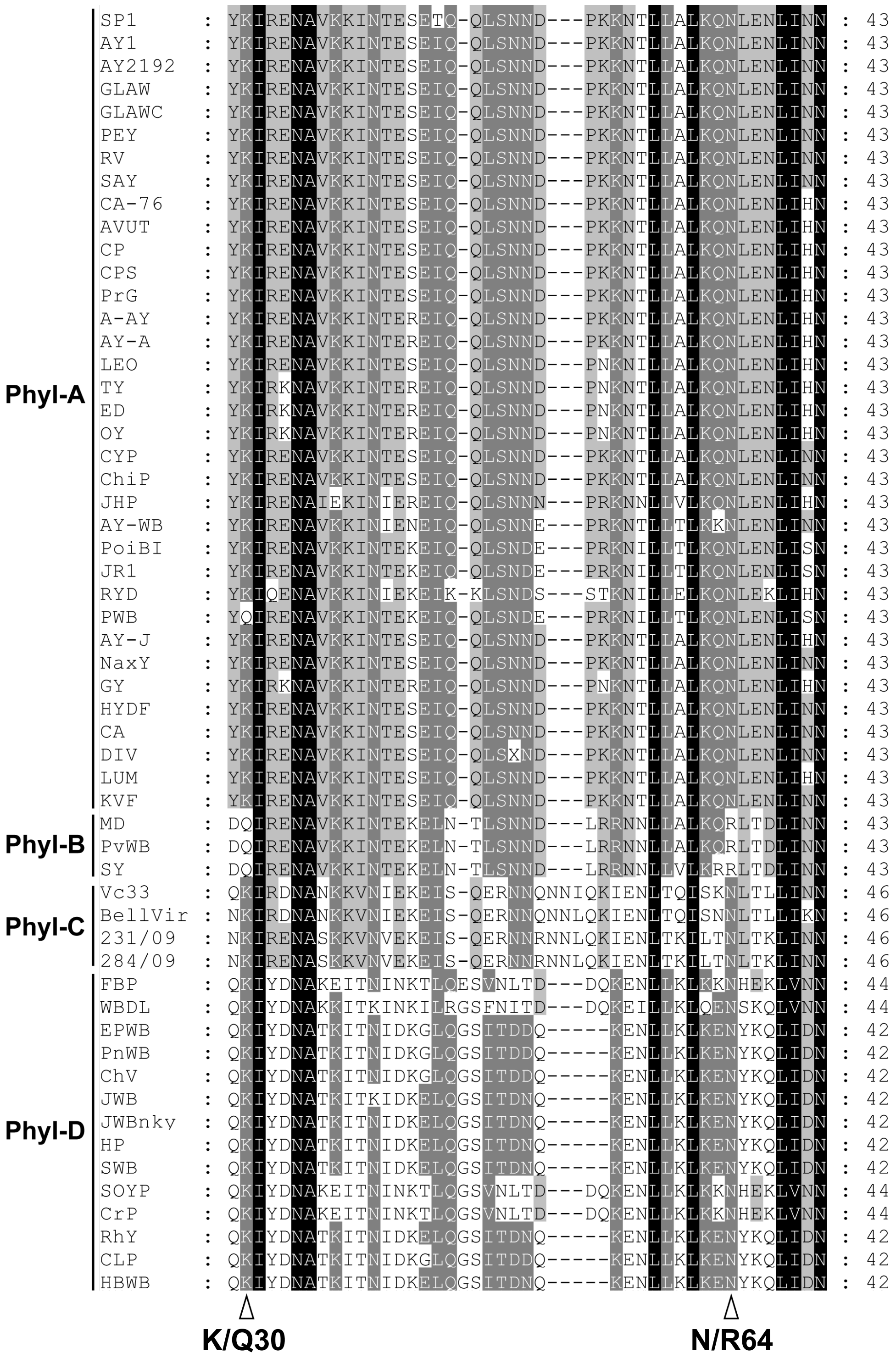

Figure S8. Alignment of protein sequences of the phyllogen family between primers PHYL-F/R used in this study.
Partial amino acid sequences of the phyllogen family between primers PHYL-F/R were aligned using the MUSCLE algorithm. Light gray, dark gray, and black shading indicate more than 60%, more than 80%, and 100% consensus in each column, respectively. Open arrowheads indicate highly conserved residues, except for the phyl-B group (K/Q30 and N/R64, numbering based on $PHYL1_{OY}$ excluding signal peptide). Three α-helix truncated phyllogens ($PHYL1_{ASHy2}$, $PHYL1_{PYR}$, and $PHYL1_{WBD}$) and two partial phyllogens ($PHYL1_{MA1}$ and $PHYL1_{NJAY}$) were excluded.
