# Supplementary material for: Functional variation in phyllogen, a phyllody‐inducing phytoplasma effector family, attributable to a single amino acid polymorphism
Source: Mol Plant Pathol. 2020 Aug 19;21(10):1322–36. doi: 10.1111/mpp.12981 (PMC7488466; doi:10.1111/mpp.12981)
Supplement: Supplementary file 9 — Figure S9 [file MPP-21-1322-s009.pdf]

Figure S9

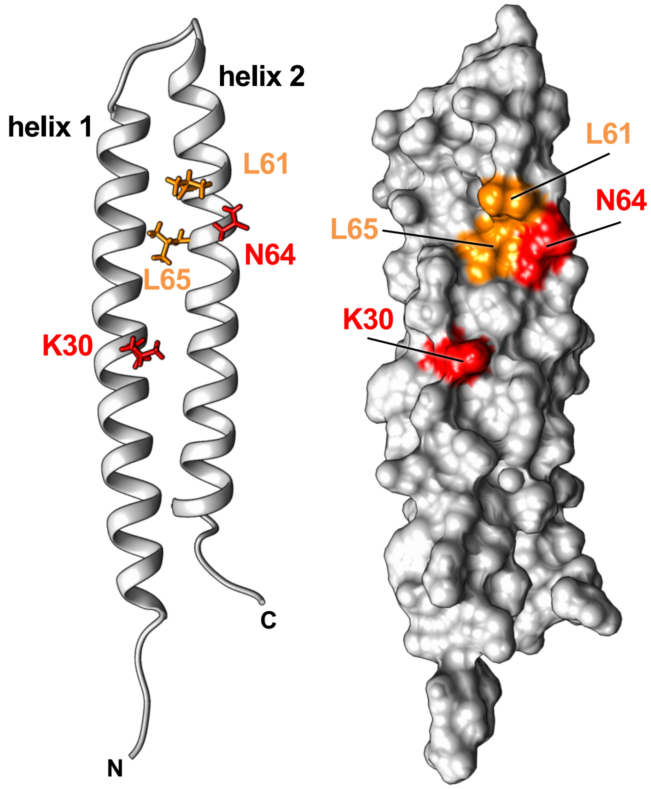

Figure S9. Positions of residues important for interacting with MTFs on the PHYL1$_{OY}$ structure. The ribbon diagram (left) and surface structure (right) representation of PHYL1$_{OY}$ (PDB ID: 6JQA). Two conserved hydrophilic residues (K30 and N64) except for all members of the phyl-B group are shown as red sticks/patches. Two hydrophobic residues involved in the interaction of PHYL1$_{PnWB}$ with SEP3 (L61 and L65 based on PHYL1$_{OY}$; Liao *et al*., 2019) are shown as orange sticks/patches.
