# Supplementary material for: Functional variation in phyllogen, a phyllody‐inducing phytoplasma effector family, attributable to a single amino acid polymorphism
Source: Mol Plant Pathol. 2020 Aug 19;21(10):1322–36. doi: 10.1111/mpp.12981 (PMC7488466; doi:10.1111/mpp.12981)
Supplement: Supplementary file 10 — Figure S10 [file MPP-21-1322-s010.pdf]

Figure S10

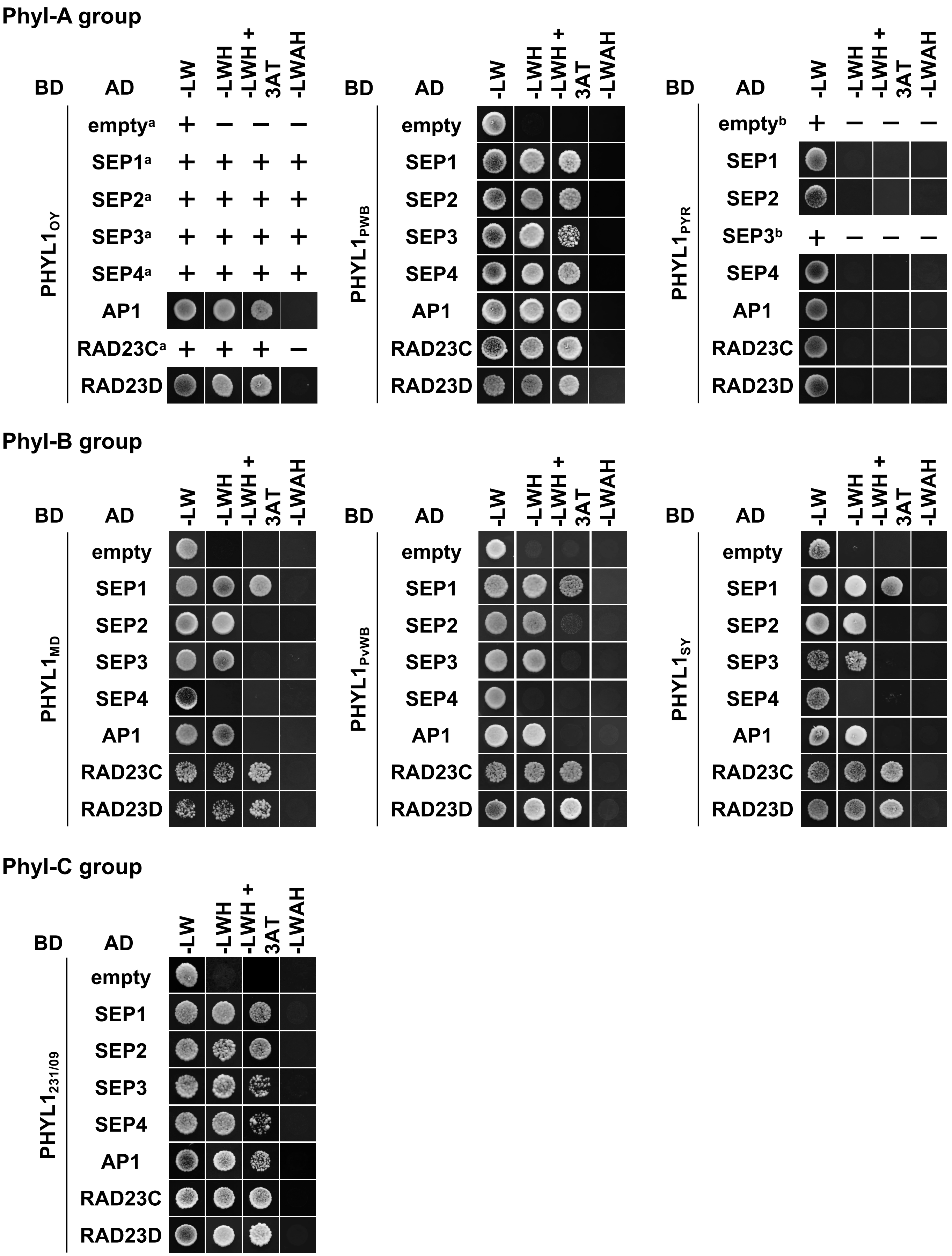

Phyl-A group
Phyl-B group
Phyl-C group
BD
AD
-LW
-LWH
-LWH + 3AT
-LWAH
PHYL1OY
PHYL1PWB
PHYL1PYR
PHYL1MD
PHYL1PvWB
PHYL1SY
PHYL1231/09
empty[a]
SEP1[a]
SEP2[a]
SEP3[a]
SEP4[a]
RAD23C[a]
empty[b]
SEP3[b]
empty
SEP1
SEP2
SEP3
SEP4
AP1
RAD23C
RAD23D

Figure S10 continued

Phyl-D group

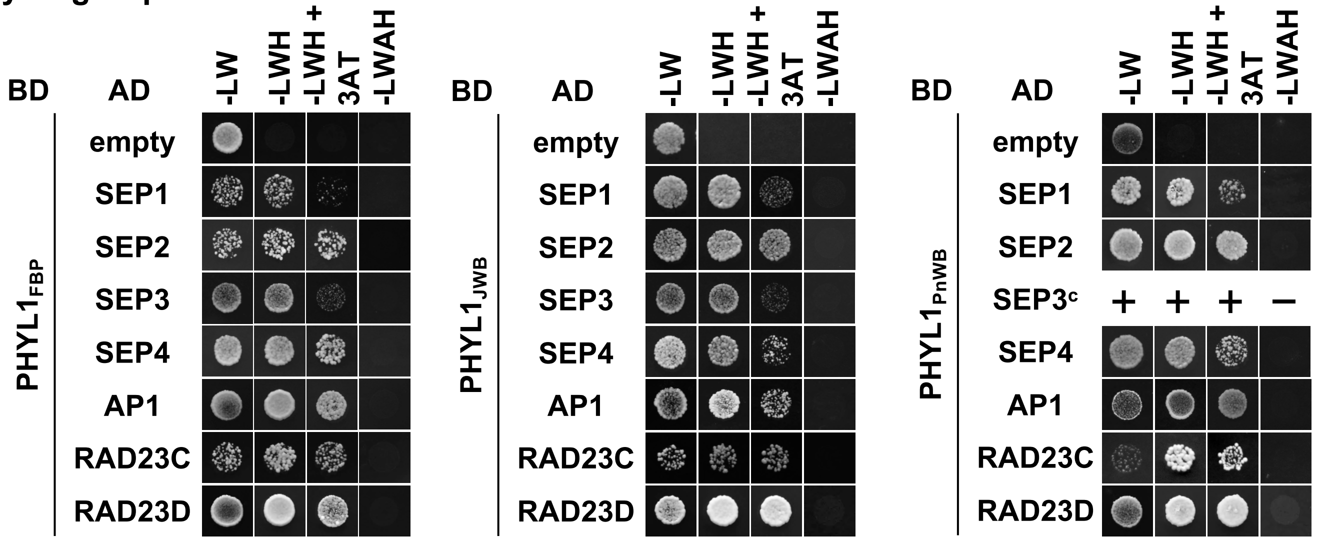

PHYL1$_{SY}$ mutants

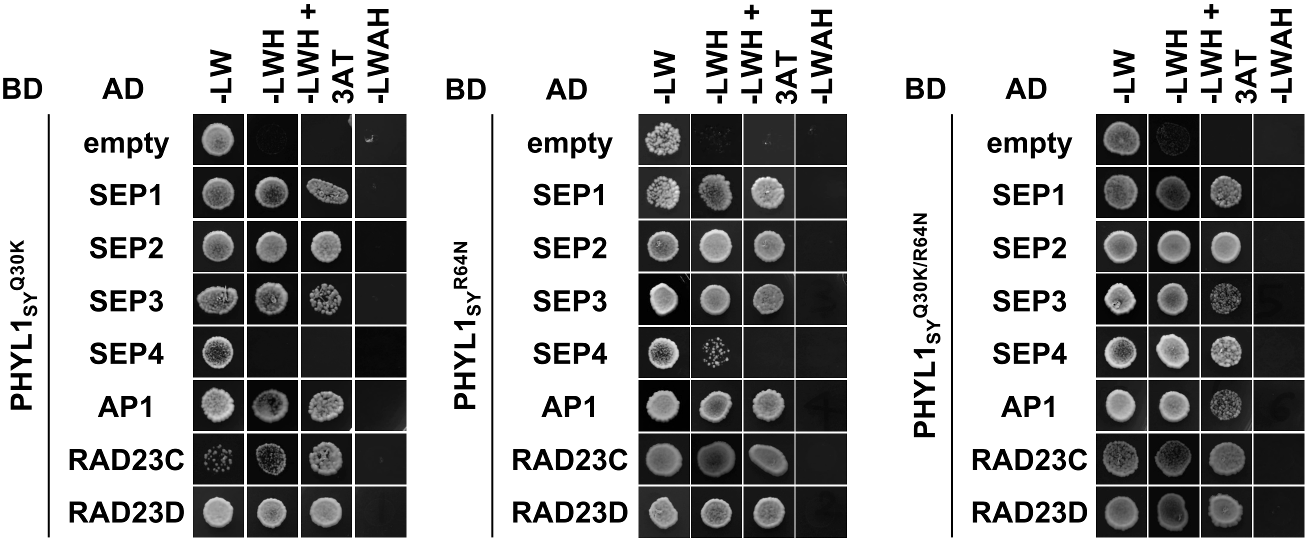

Figure S10. Growth of yeast on selective media in Y2H analyses.
An evaluation of protein interactions is summarized in Table 1. Several results indicated as + (yeast grew on selective media) or – (yeast did not grow on selective media) were previously reported in [a] Iwabuchi *et al*. (2019), [b] Maejima *et al*. (2014), or [c] Kitazawa *et al*. (2017).
